# Serial intravital 2-photon microscopy and analysis of the kidney using upright microscopes

**DOI:** 10.3389/fphys.2023.1176409

**Published:** 2023-04-24

**Authors:** Donato Sardella, Anders M. Kristensen, Luca Bordoni, Hanne Kidmose, Ali Shahrokhtash, Duncan S. Sutherland, Sebastian Frische, Ina Maria Schiessl

**Affiliations:** ^1^ Department of Biomedicine, Aarhus University, Aarhus, Denmark; ^2^ Interdisciplinary Nanoscience Center, Aarhus University, Aarhus, Denmark

**Keywords:** intravital microscopy (IVM), image denoising, animal holder, deep learning, image registration, bioimage analysis, 3D printing, multiphoton microcopy

## Abstract

Serial intravital 2-photon microscopy of the kidney and other abdominal organs is a powerful technique to assess tissue function and structure simultaneously and over time. Thus, serial intravital microscopy can capture dynamic tissue changes during health and disease and holds great potential to characterize (patho-) physiological processes with subcellular resolution. However, successful image acquisition and analysis require significant expertise and impose multiple potential challenges. Abdominal organs are rhythmically displaced by breathing movements which hamper high-resolution imaging. Traditionally, kidney intravital imaging is performed on inverted microscopes where breathing movements are partly compensated by the weight of the animal pressing down. Here, we present a custom and easy-to-implement setup for intravital imaging of the kidney and other abdominal organs on upright microscopes. Furthermore, we provide image processing protocols and a new plugin for the free image analysis software FIJI to process multichannel fluorescence microscopy data. The proposed image processing pipelines cover multiple image denoising algorithms, sample drift correction using 2D registration, and alignment of serial imaging data collected over several weeks using landmark-based 3D registration. The provided tools aim to lower the barrier of entry to intravital microscopy of the kidney and are readily applicable by biomedical practitioners.

## 1 Introduction

Intravital microscopy (IVM) of mouse and rat organs is a powerful experimental approach that provides quantitative physiology data paired with three-dimensional structural information at subcellular resolution (Vaghela et al., 2021). IVM of the kidneys was pioneered by Steinhausen et al. who leveraged IVM and computerized data analysis to provide quantitative information on the renal secretion of a Sulfonefluorescein dye (Steinhausen et al., 1976). The first application of multiphoton excitation (MPE) for IVM of the kidney (Dunn et al., 2002; Kang et al., 2006a) has revolutionized the field by substantially increasing optical tissue penetration. This enabled the visualization of previously inaccessible structures *in vivo* and allowed novel approaches for quantitative assessment of single nephron function (Kang et al., 2006b; Sandoval and Molitoris, 2008; Peti-Peterdi and Sipos, 2010; Nakano et al., 2012; Hall et al., 2013; Schiessl and Castrop, 2013; Engbjerg et al., 2022).

These early works employed acute surgical preparations, limiting the ability to track tissue changes within the same animal for longer than a few hours at a time. Approximately a decade ago, Ritsma et al. overcame this barrier by introducing Abdominal Imaging Windows (AIW) for IVM of abdominal organs, which demonstrated a viable approach toward long-term imaging of the same tissue regions (Ritsma et al., 2012; Ritsma et al., 2013). Schiessl et al. (2018), first implemented AIWs for longitudinal tracking of single renal cells, which has proven successful in multiple following kidney IVM studies (Shroff et al., 2019; Schiessl et al., 2020; Zhang et al., 2020; Desposito et al., 2021; Arndt et al., 2022; Bordoni et al., 2023).

Decoupling the organ from breathing movements is of paramount importance for IVM of abdominal organs. The most common approach for kidney IVM involves the use of inverted microscopes. Thus, the kidney (externalized or imaged through the AIW) is imaged from below, with the animal turned on its side to exploit the animal’s body weight pressing down and passively stabilizing the organ (Dunn et al., 2002; Ritsma et al., 2012; Schiessl et al., 2018; Desposito et al., 2021; Polesel et al., 2022). Upright microscopes are less commonly employed for IVM of the kidney and abdominal organs, as uncompensated breathing movements prevent direct imaging of the AIW-implanted or externalized organs. To circumvent this problem, Dunn et al. (Dunn et al., 2012) demonstrated the application of kidney cups for upright IVM of the kidney, which are typically used for renal micropuncture or other experimental approaches to measure kidney function (Vallon, 2009; Palygin et al., 2015; Shimada et al., 2022). Furthermore, upright microscopes have been converted to an inverted configuration by mounting commercially available lens inverters and placing the animal onto secondary stages laid over the main one (Sandoval et al., 2018; Costanzo et al., 2022; Engbjerg et al., 2022). However, this solution may reduce detectable signal due to the presence of optical elements in the inverter (Le Grand et al., 2008). Finally, several studies reported the use of custom-made animal holders for IVM of externalized (Jorch et al., 2019; Binz-Lotter et al., 2020) and AIW-implanted kidneys (Kessel et al., 2020; Arndt et al., 2022; Grimm et al., 2022; Kroeger et al., 2022; Bordoni et al., 2023), respectively. However, these studies did not include detailed descriptions of their custom-made stabilization tools, preventing easy adaptation by other researchers.

Mechanical tissue stabilization for IVM can markedly reduce, but not fully prevent movement-induced sample drift during live imaging (Vinegoni et al., 2014). Sample drift changes the relative position of structures of interest within the frames over time. This complicates fluorescence intensity quantification and introduces warping appearance of 3D rendered structures recorded over multiple focal planes. Image registration algorithms, however, can greatly ameliorate these issues when applied post-acquisition (Lowe, 2004; Soulet et al., 2013; Dunn et al., 2014; Parslow et al., 2014; Pnevmatikakis and Giovannucci, 2017).

IVM applications may require fast acquisition rates and/or low illumination powers to record fast biological processes, such as renal filtration or to prevent tissue damage during repeated laser-exposure. However, such imaging regimens may result in data with low signal to noise ratio (SNR) due to the presence of noise with Poisson-Gaussian statistical distribution (Zhang et al., 2007). Noise prevents accurate reconstruction of fine biological structures and increases the difficulty of downstream image analysis. Thus, several processing pipelines implement a denoising algorithm prior to image registration and/or segmentation (Arganda-Carreras and Andrey, 2017; Pnevmatikakis, 2019). Denoising algorithms can restore the original signal with varying degrees of efficacy (Dabov et al., 2007; Luisier et al., 2011; Makitalo and Foi, 2013; Meiniel et al., 2018). More recently, deep learning neural networks have been applied to this purpose, demonstrating impressive results (Krull et al., 2019; Laine et al., 2021).

Lastly, serial imaging data of the same biological structures over time adds further complexity to analysis pipelines. Sequentially acquired stacks are typically opened in a side-by-side fashion to compare equivalent focal planes and biological structures and to assess potential changes. This process can be time consuming and subject to biases when sample orientation or morphology varied throughout individual imaging sessions. Equivalent structures (e.g., renal tubules) may be subject to different optical sectioning and appear tilted, translated, or rotated in different stacks. This forces the researcher to scroll throughout several slices to find local similarities, which can lead to quantification errors. Careful animal preparation and mounting can greatly reduce this problem but will eventually prove insufficient in the presence of severe injury-induced tissue remodeling. Volumetric registration is a powerful approach for aligning biological structures in sequentially acquired datasets in a three dimensional space and is a common operation in medical imaging to ease analysis and interpretation of longitudinal patient data (Maes et al., 2003).

This manuscript aims to make IVM techniques of abdominal organs, in particular the kidney, more accessible to the research community. As inverted 2-photon microscopes are not always available, we present a novel and readily applicable image setup for (serial) IVM of abdominal organs on potentially any upright microscope capable of fitting a small rodent between the stage and the objective lens. Furthermore, we apply and outline detailed image processing steps for image denoising, sample drift correction and volumetric image registration that can be executed with the free image processing programs, ImageJ or FIJI. Lastly, we developed and provide a new plug-in for FIJI, which allows for easy and end-user friendly implementation of the presented processing tools on IVM datasets.

## 2 Methods

### 2.1 Winged abdominal imaging window (wAIW)

**FIGURE 1:**
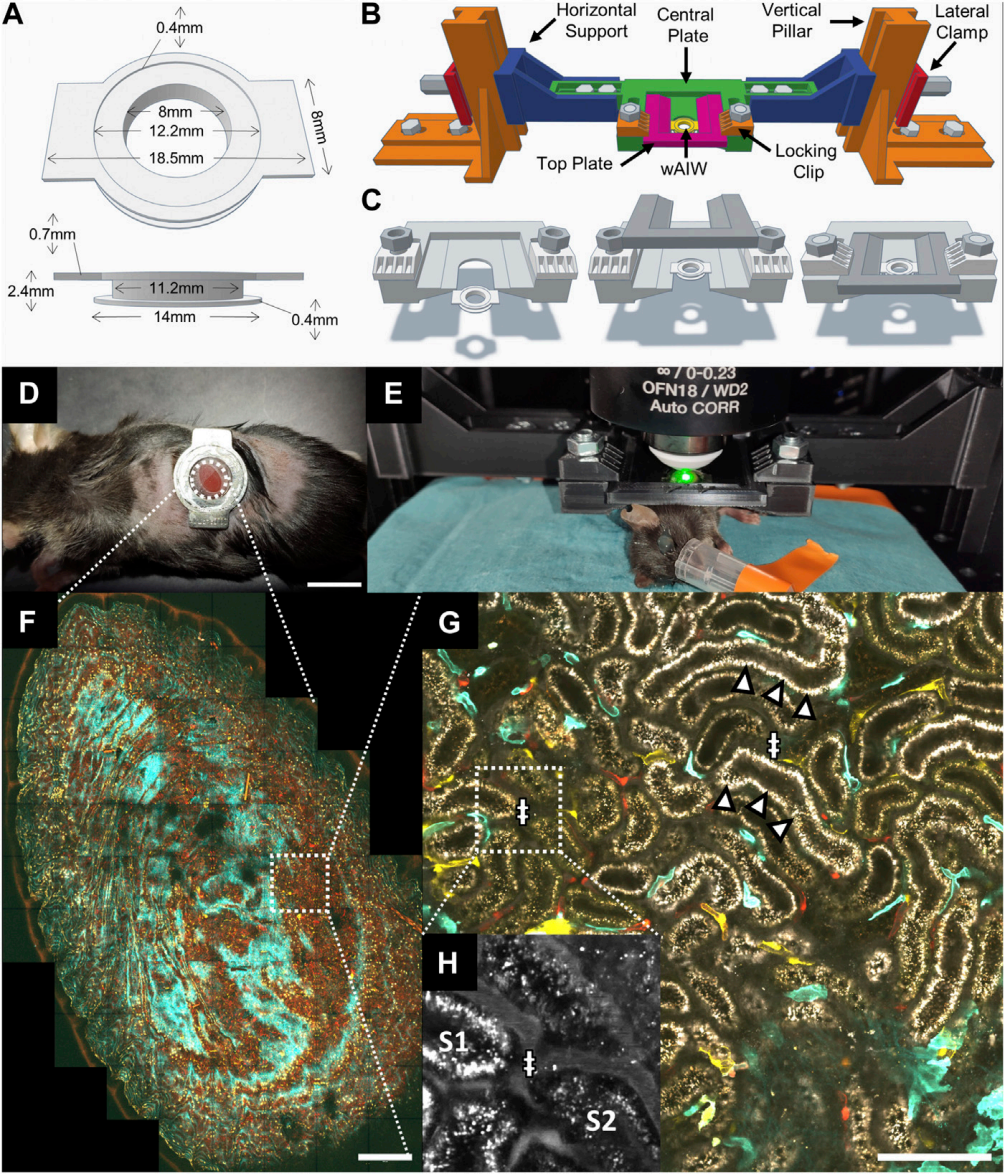
Intravital microscopy setup for upright imaging of the kidney across mm and sub-µm scales with a custom 3D printed animal holder. **(A)** 3D model and dimensions of a winged abdominal imaging window (wAIW) for serial imaging made from titanium. **(B)** wAIW slotted into a custom 3D-printed animal holder. The animal holder can be assembled from indicated parts that were printed separately on a consumer-grade 3D printer. **(C)** Individual steps of wAIW mounting. The wAIW gets slotted into the central plate of the animal holder and is locked into place by a top plate fixated through 2 lateral clips and 2 M6 bolts and nuts. **(D)** Anesthetized mouse after wAIW implantation showing correct orientation of the wings (facing dorsally and ventrally) to prevent hindlimb hindrance. Note the attached kidney surface to the coverslip. Scale bar = 10 mm. **(E)** Experimental setup for intravital microscopy of the kidney or other abdominal organs with an upright 2-photon microscope. The anesthetized mouse is mounted to the animal holder and placed on a heating pad on the microscope stage. **(F)** Maximum intensity projection of an intravital 2-photon microscopy 3D atlas scan acquired in a Cdh5-Confetti mouse kidney (Table 1) illustrates the potential of intravital microscopy to image mm-scale tissue regions. Scale bar = 500 µm. **(G)** High-resolution view (4096 × 4096 px, 124 nm/px) of a Cdh5-Confetti mouse provides paired morphological and functional readouts. Endothelial cells expressing the Brainbow2.1 reporter system can be identified through 10 different color combinations (Table 1). A Setau-665-BSA dye (gray) labels the lumen of peritubular capillaries (ǂ) and apical albumin reabsorption in renal proximal tubules (arrowheads). Scale bar = 100 µm. **(H)** Zoomed-in inset of Setau-665-BSA detection channel (displayed in grey) demonstrates blood plasma labeling in peritubular capillaries (ǂ).

For intravital imaging of abdominal organs on an upright microscope, we developed winged abdominal imaging windows (wAIWs) (Figure 1A), through modification of a previously published abdominal imaging window design (Ritsma et al., 2013). wAIWs were custom-made from medical-grade titanium and consisted of 2 rings with an outer diameter of 14 mm and an inner diameter of 8 mm, each separated by a groove with a height of 1.3 mm and a depth of 2.2 mm. Two rectangular wings (2 mm × 8 mm) with rounded edges protruded from the upper ring on opposite sites, resulting in a maximum width of 18.5 mm. The upper ring further hosted a round inset of 12.2 mm in diameter and 0.4 mm in depth to fit a round glass coverslip (12 mm diameter, Menzel-Glaeser, Germany), which was attached to the wAIW with cyanoacrylate glass glue (Loctite-Henkel, Germany) as described before (Schiessl et al., 2020). Three-dimensional models of the wAIW are included in Supplementary Material as STL models.

### 2.2 Optional wAIW passivation protocol

To enhance long-term visual clarity of wAIW implants, coverslips can be processed with oxidation-stable antifouling brushes of 5 kDa Poly-2-methyl-2-oxazoline (PMOXA) (Chen et al., 2019) with predefined spacing grafted on a polyacrylamide (PAA) backbone. High-density PMOXA brushes were grafted to the coverslip surface using high-affinity electrostatic amine linker and covalent silane linkers designed on PAA backbone for long-term stability (Konradi et al., 2012).

To prepare the Borosilicate glass coverslips for the deposition of the antifouling polymer, they were cleaned by ultrasonication in acetone and 96% ethanol, followed by drying under N2 stream. Each side of the coverslips was plasma cleaned for 5 min using a Diener Femto Plasma Etcher at 100 W at ≈35 mTorr, and an O2 flow of 50 SCCM to activate the surface for attachment of covalent silane linkers. The plasma-cleaned coverslips were immediately transferred to a sterile Petri dish, moved to a sterile environment in a laminar airflow bench, and were transferred to a sterile 50 mL Falcon tube containing 0.2 μm filtered solution of 0.1 mg/mL PAA-g-PMOXA (NH2, Si), (SuSoS AG - Switzerland) dissolved in 1 mM HEPES buffer at pH 7.4. According to an adapted protocol from Ogaki et al. (Ogaki et al., 2012), the tubes were incubated for 24 h in a water bath at 80°C to achieve ultra-dense coating and optimize the longevity of the antifouling coating. After 24 h of incubation, the coverslips were carefully removed from the solution, rinsed with an excess of 0.2 μm filtered deionized H2O, and dried under N2 stream. For wAIW preparation, the coverslip side facing upwards during drying was oriented to finally face inside the abdomen of the animal after implantation.

### 2.3 3D printed animal holder for intravital imaging on upright scopes

For imaging on upright microscopes, the wAIW was mounted to a 3D printed animal holder. The animal holder was designed in Tinkercad (Autodesk, United States) and exported in STL (Standard Triangle Language) format. The STL files were then prepared for 3D printing with the open-source software Cura (Ultimaker, Netherlands) using a layer height of 0.2 mm, 5 perimetral walls, and a 60% infill density with a “Grid” pattern. Models were finally manufactured with an Ender 3 pro 3D printer (Creality, China), mounting a 0.4 mm diameter extrusion nozzle and using a PLA (Poly-Lactic Acid) polymer filament. Printing settings included 50 mm/s extrusion speed, 200°C nozzle temperature and 60°C for the printer bed. The animal holder consisted of two vertical pillars which allowed anchoring to the microscope stage with M6 screws, two height-adjustable horizontal supports, two lateral clamps to lock the supports to the pillars and a horizontal central plate for slotting of the wAIW (Figure 1B). Finally, a top plate covered the slotted wAIW, which was stabilized by 2 M6 screws pushing down two locking clips (Figure 1C). To ensure reproducibility of the holder, all the components are included in the Supplementary Material as three-dimensional models in STL file format.

### 2.4 Animals

Data presented in this manuscript was acquired from multiple transgenic reporter mouse strains with C57BL/6 genetic background. Male and female mice between 8 and 24 weeks of age were used. Mice were bred and housed at the animal facility at the Department of Biomedicine, Aarhus University (Aarhus Denmark), or the Center of Functionally Integrative Neuroscience at the Preclinical Research Facility at Aarhus University Hospital. After weaning, mice were kept in groups of two to five subjects per cage (Technoplast GM500 or GM9000), provided with *ad libitum* access to food and water, a 12-h light/darkness cycle, an environmental temperature of 21°C, and the presence of behavioral enrichment elements, including cardboard Shepherd Shacks (Shepherd Specialty Papers, United States). Cages were cleaned once a week.

A detailed overview of transgenic fluorescent reporter strains used, and procedures performed is provided in Table 1. All experiments were performed in accordance with Danish law and under the authorization of the Animal Experiments Inspectorate with permit number 2020-15-0201-00443.

**TABLE 1:** Animal strains, procedures, and imaging parameters.

Figure	Mouse strain	Experimental procedure	Fluorescent proteins/dyes	Imaging setup	Fluorescence detection and look up tables
1 Intravital microscopy setup	**Cdh5-Confetti (**C57BL/6-Tg (Cdh5-cre/ERT2) 1Rha X B6.129P2-Gt (ROSA)26Sortm1(CAG-Brainbow2.1)Cle/J (C57BL/6)**)**	AIW implantation Retroorbital dye injection Imaging on day 0 after AIW	Homozygous expression of membranous CFP, nuclear GFP, cytosolic YFP and RFP in endothelial cells, resulting in 10 different color-combinations Desposito et al. (2021) Setau-665-BSA (plasma labeling)	Olympus FVMPE-RS λ_ex_ 940 nm	Ch1—Gray (660–750 nm) Ch2—Red (570/645 nm) Ch3—Yellow (520–560 nm) Ch4—Cyan (460–500 nm)
2 and S1 Denoising validation dataset	**Cdh5-tdTomato (**C57BL/6-Tg (Cdh5-cre/ERT2) 1Rha X B6.Cg-Gt (ROSA) 26Sortm9 (CAGtdTomato) Hze/J (C57Bl/6)**)**	AIW implantation Ischemia Reperfusion Injury (IRI) Imaging on day 1 after IRI and AIW	tdTomato in endothelial cells	Olympus FVMPE-RS λ_ex_ 860 nm	Ch1—Red (570–606 nm) Ch2—Green (510–530 nm) Ch3—Blue (425–475 nm)
3 2D-registration Tseries	**BL/6** (C57BL/6NTac)	AIW implantation Carothid cannulation for dye bolus injection Imaging on day 0 after AIW	FITC-Dextran 4 kDa (fluid phase marker for bolus tracking) AlexaFluor594-BSA (plasma labeling)	Brucker Ultima Investigator λ_ex_ 750 nm	Ch1—Red (570–620 nm) Ch2—Green (500–550) Ch3—Blue (435–485)
4 2D-registration Zstack	**Cdh5-Confetti (**C57BL/6-Tg (Cdh5-cre/ERT2) 1Rha X B6.129P2-Gt (ROSA) 26Sortm1 (CAG-Brainbow2.1) Cle/J (C57BL/6)**)**	AIW implantation Imaging on day 2 after AIW	Homozygous expression of membranous CFP, nuclear GFP, cytosolic YFP and RFP in endothelial cells, resulting in 10 different color-combinations Desposito et al. (2021)	Brucker Ultima IV λ_ex_ 920 nm	Ch1—Red (570–620 nm) Ch2—Green (500-550 Ch3—Blue (435–485)
5 3D-registration	**Cdh5-tdTomato (**C57BL/6-Tg (Cdh5-cre/ERT2) 1Rha X B6.Cg-Gt (ROSA) 26Sortm9 (CAGtdTomato) Hze/J (C57Bl/6)**)**	AIW implantation Ischemia Reperfusion Injury (IRI) Imaging on days 1, 7 and 17 after IRI and AIW	tdTomato in endothelial cells	Olympus FVMPE-RS λ_ex_ 860 nm	Ch1—Red (570–606 nm) Ch2—Green (510–530 nm) Ch3—Blue (425–475 nm)
S2 3D-registration	**Cdh5-Confetti (**C57BL/6-Tg (Cdh5-cre/ERT2)1Rha X B6.129P2-Gt (ROSA) 26Sortm1 (CAG-Brainbow2.1) Cle/J (C57BL/6)**)**	AIW implantation Imaging on days 1 and 7 after AIW	Homozygous expression of membranous CFP, nuclear GFP, cytosolic YFP and RFP in endothelial cells, resulting in 10 different color-combinations (Desposito et al., 2021)	Brucker Ultima IV λ_ex_ 860 nm	Ch1—Red (570–606 nm) Ch2—Green (510–530 nm) Ch3—Blue (425–475 nm)

### 2.5 Abdominal imaging window implantation and postoperative care

Anesthesia was induced with isoflurane gas at a concentration of 4% and maintained at 1.5%–2% (flow rate: 1 L/min, 50/50% Air/O_2_). After anesthesia induction, animals received 3.3 μL/g BW of Buprenorphine (Labiana Life Sciences, Spain, stock: 0.3 mg/mL) i. p. Surgical implantation of the wAIW was performed as described previously (Ritsma et al., 2013; Alieva et al., 2014; Schiessl et al., 2020). While attaching the kidney to the coverslip of the wAIW using cyanoacrylate glass glue (Loctite-Henkel, Germany), correct orientation of the wAIW is critical. Thus, the 2 wings of the wAIW need to be faced dorsally and ventrally, to avoid interference with the hindlimb of the mouse (Figure 1D). Circulating volume was maintained with 10 μL/g BW sterile 0.9% saline i. p. once per hour. After the implantation procedure, mice received 4 μL/g BW meloxicam (Boehringer Ingelheim Vetmedica GmbH, Germany, stock: 0.25 mg/mL) s. c., were withdrawn from anesthesia, and allowed to recover.

Follow-up analgetic treatment of mice was maintained for 3 days after the surgery. Mice received drinking water with 1 mL buprenorphine (stock solution: 0.3 mg/mL) diluted in 33 mL of water. This volume was found to be adequate for up to 4 animals per cage. Moreover, mice received 4 μL/g BW of meloxicam (stock: 0.25 mg/mL) s. c. for 3 days.

Mice which underwent wAIW implantation were monitored daily to ensure absence of animal distress/discomfort, infections, and tissue necrosis surrounding the implant. Mice were allowed to survive for up to 6 weeks after the AIW surgery.

### 2.6 Serial intravital two-photon microscopy of the kidney

Intravital microscopy was performed on live anesthetized mice mounted on our custom 3D printed holder.

Prior to imaging sessions, anesthesia was induced with 4% isoflurane mixed in air and maintained with 1.3%–1.8% isoflurane (flow rate: 75–105 mL/min) using a SomnoSuite apparatus (Kent Scientific, United States). wAIW-implanted mice were mounted on the 3D printed animal holder fixed to the stage of the upright 2-photon microscope (Figures 1B–E) and placed on a heating pad connected to the SomnoSuite for monitoring and maintenance of physiological body temperature. Eye ointment (Viscotears, Chem. Pharm. Fabrik GmBH, Germany) was applied to ensure cornea hydration and circulating volume was maintained by hourly injection of sterile 0.9% saline (10 μL/g BW) i. p. (Rhodes, 2017). During the imaging session, mice were constantly monitored with an infrared camera (ELP, China).

Prior to image acquisition, the following fluorescent dyes were injected retro-orbitally after application of local anesthetic solution (Xylocain, AstraZeneca, UK, stock: 5 mg/mL): To label blood plasma, 1–2 µL/g BW of purified stock solutions of either AlexaFluor-594-BSA (Thermo Fisher Scientific, United States), AlexaFluor-680-BSA (Thermo Fisher Scientific, United States), or Setau-665 (SETA Biomedicals, United States) conjugated to Bovine Serum Albumin (Thermo Fisher Scientifc, United States) were used. For bolus tracking experiments, 10 µL of a 40 mg/mL FITC-Dextran 4 kDa (TdB Labs, Sweden) solution was injected through a jugular vein catheter. See Table 1 for detailed experimental overview.

Intravital imaging data were acquired on three different upright 2-photon microscopes: a Bruker Ultima IV (Bruker, United States), a Bruker Ultima Investigator Plus (Bruker, United States) and an Olympus FVMPE-RS (Olympus, Japan). The Bruker systems were equipped with 4 GaAsP detectors and each mounted an XLUMPFL20XW (Olympus, Japan) water immersion objective (×20, NA = 1.0, WD = 2 mm). Excitation was provided by a Chameleon Ultra II Ti-Sapphire laser (Coherent, United States). The Olympus FVMPE-RS was equipped with 2 multialkali PMTs and 2 GaAsP detectors and mounted an integrated Olympus FV30-AC25 W objective (×25, NA = 1.05, WD = 2 mm). Excitation was provided by a Mai Tai HP DS-OL 2-photon laser system (Spectra Physics, United States).

Unless specified differently, images were acquired with a galvanometer scanner using 2 µs dwell time and saved with 512 × 512 pixels image resolution. Laser power and detector gains were adjusted to avoid signal saturation and/or clipping while minimizing illumination power to avoid phototoxicity or bleaching. For a detailed overview of excitation and emission-detection settings applied in different experiments, please refer to Table 1.

Serial imaging of the same tissue regions was facilitated through creation of atlas scans of the attached kidney surface to the wAIW (Figure 1F). During the first imaging session, the outer contour of the tissue adhering to the coverslip was identified and imaged as a tiled scan to reconstruct and map the gross shape and profile of the accessible renal tissue in the wAIW. In the following imaging sessions, atlas scans were used as visual aid to assist navigation and identification of the same fields of view over the course of multiple weeks.

#### 2.6.1 Preparation of fluorophores for blood plasma labeling

AlexaFluor-BSA dyes (Thermo Fisher Scientific, United States) were resuspended in phosphate buffer saline (PBS, pH 7.4) to a concentration of 2.5 mg/mL, pipetted in Nanosep 30 K Omegaspin filter spin columns (Pall Corporation, United States) and centrifuged for 5 min at 14,000 rpm to discard free AlexaFluor dye molecules in the solution. The supernatant was then collected and diluted in 1.5 mL PBS resulting in a solution with an approximate concentration of 2.5 mg/mL that was aliquoted and stored at −19°C until use.

SeTau-665-BSA was prepared from 1 mg of dry Setau-665-NHS ester powder (SETA BioMedicals PN K9-4119, United States) and conjugated to 20 mg of Bovine Serum Albumin (BSA, Thermo Fisher, United States). Conjugation and assessment of dye-to-protein-labeling-ratio were performed according to SETA Biomedicals technical documentation. The protocol typically yielded a total injectable volume of 1.3 mL SeTau-665-BSA with a dye-to-protein ratio of 1.0, a dye concentration of 300 µM, and a protein concentration of 16 mg/mL. After conjugation, free SeTau-665 dye molecules were removed from the solution using the same protocol as for AlexaFluor-BSA dyes.

### 2.7 Image processing

Imaging data were analyzed on a workstation PC mounting a Ryzen Threadripper 3,960X processor (AMD, United States) with 24 execution cores, 128 GB of RAM, an Nvidia 2070S GPU with 8 GB VRAM and running Windows10 (Microsoft). ImageJ (Schneider et al., 2012), FIJI (Schindelin et al., 2012) and Matlab (Mathworks, United States) were used for processing imaging data in multiple stages, beginning with denoising, followed by sample drift correction and landmark-based volumetric image registration for correlative imaging. The volumetric rendering of Figure 4 was performed in the FIJI plugin ClearVolume (Royer et al., 2015).

#### 2.7.1 Image denoising

Multiple denoising algorithms were tested and validated for their ability to reconstruct the original signal of intravital 2-photon microscopy data corrupted by mixed Poisson-Gaussian noise.

##### 2.7.1.1 Validation dataset for evaluation of denoising performance

All demonstrated denoising algorithms were applied to the same dataset (validation dataset), which was acquired in the kidney of an anesthetized mouse. Thus, an intentionally noisy 4D multichannel stack was generated through repeated imaging of the same volume over time (25 replicates) while applying high detector gains and low laser power (Table 1).

##### 2.7.1.2 Median 3D

Images were processed in FIJI using a Median 3D filter in FIJI with a σ = 2 for each dimension.

##### 2.7.1.3 Noise2Void

The deep learning neural network Noise2Void (N2V) (Krull et al., 2019) was set up in a separate FIJI instance by installing the ImageJ-Tensorflow (https://github.com/imagej/imagej-tensorflow/) and CSBDeep (Weigert et al., 2018) ImageJ plugins. N2V is a self-supervised deep learning architecture that does not require clean target images for reference and training. Therefore, training was directly performed on the validation dataset (noisy data) that was also later used to test N2V performance. During training, N2V splits the image into small patches and predicts the intensity value of a blind spot pixel contained within a patch. This process is repeated for multiple image patches until the network performance gradually improves with each training epoch (Krull et al., 2019).

The CSBDeep plugin provided a graphical user interface within FIJI for N2V neural network training and prediction. N2V training was performed individually for all channels of the validation dataset. Composite stacks were split into single channel stacks and saved in separate folders. Training was performed using the “N2V train on folder” command within CSBDeep.

Multiple stacks from each channel and totaling at least 1,000 slices, were used for training of two different N2V architectures:

• N2V2D: trained only on 2D image patches and corresponding to CSBDeep default settings; iterations = 200, epochs = 300, batch size = 64, patch size = 64px, neighborhood radius = 5px
• N2V3D: trained on isometric 3D image volumes; iterations = 200, epochs = 300, batch size = 16, patch size = 48px, neighborhood radius = 5px (training parameters were chosen to fit within the memory available on the Nvidia GPU that was used)

At the end of the training session, the networks from the last epoch were saved to a bioimage. io zip archive.

Finally, image denoising using trained networks was performed within CSBDeep by splitting each stack into individual channels and applying the corresponding trained N2V network with the command “N2V predict”. At prediction time, the trained N2V neural networks predicted the intensity value of each pixel in the raw noisy data based on the intensities of its neighbors, using the model obtained during training.

##### 2.7.1.4 PureDenoise

The PureDenoise (Blu and Luisier, 2007; Luisier et al., 2009; Luisier et al., 2010) algorithm was executed in ImageJ (Schneider et al., 2012) due to incompatibility of the batch execution mode with FIJI (Schindelin et al., 2012). Denoising of the validation dataset was performed for each channel individually, after splitting the original data into its constituting channels. PureDenoise was executed with automated noise estimation set to “Global”, adjacent frames = 5 and cycles = 10. Denoised stacks had 32-bit format and were then rescaled to 16-bit by rounding floating point values to unsigned integers. Finally, denoised 16-bit single channel stacks were re-assembled as composite stacks.

##### 2.7.1.5 VST-BM4D and VST-BM3D

The Gaussian denoisers BM3D (Dabov et al., 2007) and BM4D (Maggioni et al., 2013) were used to process single images or stacks with more than one slice, respectively. The two algorithms are grouped in the text under the term “BMxD”.

The BMxD denoising pipeline was split between FIJI and Matlab with temporary files written on disk to exchange data between the two programs. FIJI was tasked with batch data input/output, handling of multichannel images, and merging of denoised outputs. On the other hand, Matlab hosted the execution of BMxD algorithms in charge of denoising one channel at a time by reading the temporary file written on disk by FIJI and outputting the denoised data in a separate temporary file. This arrangement was chosen to provide a familiar interface to life scientists and exploit the wide compatibility with multiple microscopy image formats provided by the Bioformats library (Linkert et al., 2010) integrated in FIJI.

The BMxD denoising pipeline within Matlab followed the previously described strategy (Makitalo and Foi, 2011; Makitalo and Foi, 2013) to treat Poisson-Gaussian noise with Gaussian denoisers, and was broken down to 4 main steps:

• Estimation of image noise standard deviation using the algorithm from Yang&Tai (Yang and Tai, 2010) implemented by Dr. Schwemmer C. and freely available at https://www5.cs.fau.de/en/our-team/schwemmer-chris/software/index.html.
• Noise variance stabilization with a Generalized Anscombe Variance Stabilized Transform (GAT) rescaling the raw intensities to a variance equal to 1 (Starck et al., 1994; Starck, 1998).
• BMxD image filtering.
• Inversion to the GAT with a closed-form approximation of the inverse transformation (Makitalo and Foi, 2011; Makitalo and Foi, 2013).

Image noise standard deviation was estimated on a single noisy image (BM3D) or on a tiled estimation image derived from up to 10 slices evenly spaced throughout the stack (BM4D). Tiling was done to improve the accuracy of the noise estimation as previously suggested (Maggioni et al., 2012).

The estimated noise standard deviation was then used as a parameter for the GAT and applied to the noisy images resulting in data having a Gaussian noise distribution that could be processed efficiently by BMxD. After variance stabilization and prior to BMxD filtering, the intensities were then rescaled in the [0 1] interval as required by the filtering algorithm. To speed up the execution of the BM4D algorithm and distribute the computational load on multiple CPU cores, data was split in chunks of 10 frames overlapping each other by 5 frames at each extremity.

After filtering, the denoised stacks were rescaled to the original GAT stack values and a closed-form approximation of the inverse GAT was applied to further rescale the pixel intensities to the original range.

##### 2.7.1.6 Validation of denoising algorithms on intravital two-photon microscopy data

Denoising algorithms were validated by quantitative and qualitative assessment against a Ground Truth (GT) (Lemoigne et al., 2008) Zstack, which was obtained from the validation dataset (Table 1). Thus, generation of the GT was facilitated by averaging of all 25 noisy replicates of the same volume, to a single noise-free stack. Therefore, the validation dataset was imported in FIJI, and all time points were exported as separated stacks and processed for 3D registration in BigStitcher (Horl et al., 2019) as outlined in detail in Supplementary Materials. Eventually, a single noise-free GT 3D stack was obtained by averaging all 25 timepoints for each slice.

Quantitative validation of denoising performance was performed by measuring the peak signal to noise ratio (PSNR) between all the slices of the GT stack with their corresponding noisy and denoised slices (Laine et al., 2021) over the 25 time points (n = 1,125). This was achieved with the “SNR, PSNR, RMSE, MAE” ImageJ plugin (Sage and Unser, 2003) available at http://bigwww.epfl.ch/sage/soft/snr/. The analysis was performed for raw and denoised stacks on each detection channel. A statistical analysis to assess statistically significant differences between the performance of the various denoising algorithms was then performed with a non-parametric Kruskal Wallis test followed by a Dunn’s test in Graphpad Prism 8 (Graphpad, United States). The statistical tests were run on PSNR values for each channel separately.

Qualitative assessment exploited the presence of heterogeneous structures in each channel to judge the ability of denoising algorithms to efficiently recover fine structures such as thin cellular processes of tdTomato-expressing endothelial cells, small and bright lysosomes in renal proximal tubule epithelial cells and collagen fibrils.

#### 2.7.2 2D image registration

To compensate for occasional sample drifts during live imaging, as recorded in Zstacks (xy + z) and Tseries (xy + t), we performed 2D registration using the FIJI implementation of the Scale Invariant Feature Transform algorithm (SIFT) (Lowe, 2004), which was extended by the multichannel plugin available from the PT-BIOP FIJI update site (Guiet and Chiaruttini, 2022).

SIFT 2D registration operates on a single channel basis. To increase the detectable image features for successful 2D registration, a “registration channel” was generated by merging signals from all recorded detection channels of the data set in a single channel. The registration channel was created through mathematical addition of the raw values of all channels to a 32-bit registration stack. Histogram stretching of each frame over the entire bit depth range was then performed to enhance the contrast of image features with the FIJI command “Enhance Contrast” (Use slice histogram, Normalize, 1% Saturated Pixels). The registration channel was then converted and rescaled to the original bit depth and merged to the original stack before executing SIFT. The SIFT algorithm was executed with the following parameters: initial blur = 1.5, steps per scale octave = 3, minimum image size = 64, maximum image size = 512, feature descriptor size = 8 and expected transformation = Translation). Finally, after registering the stack, the registration channel was removed from the aligned stack.

Successful Tseries and Zstack registration was qualitatively evaluated. For evaluation of Tseries alignment, a Tseries was used, which recorded renal tubules after i. v. injection of FITC-4kDa-dextran (Table 1). Mean fluorescence FITC-Dextran 4kDa signal in peritubular vascular regions was measured over time, by placing manual ROIs over peritubular capillaries and then merging them to a unique ROI using a Boolean OR operation in the FIJI ROI manager. Average signal intensity over time was measured over the merged ROI. The data was then plotted in Graphpad Prism 8 (Graphpad, United States). The effect of registration was evaluated by removal of artificial drops in fluorescence due to movement artefacts and through a visual overlay of the first and last frames of the registered and unregistered datasets, respectively. Zstack (Table1) alignment was assessed through visual comparison of XZ cross-sections of the registered and unregistered stacks. Thus, the absence of wave-like image artefacts in small anatomical structures, such as apical lysosomes of proximal tubular epithelium cells and fluorescent protein-expressing endothelial cells, was used to confirm a successful registration.

#### 2.7.3 3D registration with manual landmarks

Correlation of volumetric intravital microscopy data collected over the course of up to several weeks was performed by manual landmark-based volumetric registration within the FIJI plugin BigWarp, using rigid rotation transform (Bogovic, 2016).

The registration workflow involved the visual identification of common structures between a reference (e.g., first imaging time point) and a moving stack (e.g., consecutive imaging time point). The two volumes were opened in BigWarp and landmarks were placed on corresponding renal tissue features identifiable in both stacks (Supplementary Figure S2). For placing landmarks, unique tubule shapes as outlined by autofluorescence, epithelial cell nuclei, apical lysosomes or fluorescently labelled cells could also be used. Landmark addition was performed with concurrent application to monitor the successful registration of the stacks. The registration was assessed in a side-by-side mode and a landmark number between 10 and 20 delivered satisfying results in 512 × 512 pixel datasets. A detailed step-by-step protocol is provided in Supplementary Material.

##### 2.8 Intravital microscopy processing toolbox for FIJI

For easy end-user implementation of the outlined image processing techniques, a new FIJI plugin was developed. The “Intravital Microscopy Processing Toolbox” facilitates automatic processing of image denoising with PureDenoise, Noise2Void, and BMxD, as well as drift correction using SIFT 2D image registration. The plugin allows processing of single images and stacks as well as automated batch processing of large datasets. A detailed manual on the Intravital Microscopy Processing Toolbox, installation instructions, and software codes, are provided in the Supplementary Material.

## 3 Results

### 3.1 Winged abdominal imaging windows

To expand the pool of available imaging systems for intravital imaging of the kidney and other abdominal organs to upright microscopes, we added lateral wings to the previously described AIWs (wAIW, Figures 1A, D) (Ritsma et al., 2013). wAIW implantation did not demand further changes to the previously published surgical protocol (Ritsma et al., 2013; Alieva et al., 2014; Schiessl et al., 2020). wAIW-mice did not display overt behavioral changes and signs of distress. Optimal positioning of the titanium ring relative to the rib cage and with the wings facing dorsally and ventrally, respectively, avoided hindrances to hind leg motion and the insurgence of skin irritation due to rubbing with the wings (Figure 1D). Our AIW design was successfully tested on mice ranging from 15 to 35 g of body weight, thus enabling a wide variety of animal models to be investigated. Removal of any hard plastic housing and environment-enriching elements (e.g., tunnels) with narrow passages and crevices prevented wAIW-animals from getting stuck or suffering injuries. Soft cardboard-based housing and increased soft bedding material were found to be safe replacements. No further changes to animal cages (e.g., feeding through) were necessary.

### 3.2 3D printed animal holder for serial intravital microscopy imaging

Our custom 3D printed animal holder was designed to mount live mice (Figure 1E) and stabilize abdominal organs during intravital imaging while maintaining constant tissue orientation over time for serial imaging.

Manufacturing of the 3D printed holder produced good results on basic FDM (Fused Deposition Modeling) 3D printers loaded with common PLA filament. Load-bearing parts like the supporting pillars, clamps and the horizontal support bar possessed sufficient rigidity deriving from the increased wall count and high-density print infill. The holder could be quickly assembled and disassembled on the microscope stage, providing easy implementation on shared equipment within imaging facilities. The anchoring feature of the holder through 2 M6 screws on the vertical pillars, allowed repeated fixing of the holder on the microscope stage in consistent orientation, minimizing rotations of serial imaging data collected on different days. The usability of the 3D printed holder was tested on three separate upright imaging systems, each provisioned with different stages. The holder was found compatible with multiple objectives, including state-of-the-art long-distance objectives, optimized for intravital imaging (Bordoni et al., 2023), as well as with high magnification, low working distance lenses, optimized for confocal microscopy (Grimm et al., 2022).

The wings of the wAIW allowed solid attachment to the 3D printed animal holder. Mounting an anesthetized AIW-implanted mouse was quickly accomplished by following 3 easy steps (Figure 1C). Once locked in place, the tissue attached to the wAIW was largely decoupled from breathing movements. Axial shifts of the tissue were also largely reduced, while slight lateral drifts could sporadically occur. Tissue stabilization, achieved through mounting of the wAIW in the animal holder, was sufficient to acquire high-resolution images (4096 × 4096px) covering large field of views (Figure 1G).

Serial imaging of the same field of views over the span of several weeks was facilitated through referencing of atlas scans (Figure 1F) acquired on different imaging days. Unique tissue features (e.g., specific renal nephron segments or distinct patterns of fluorescent protein-expression in transgenic animals) could be identified within the tissue and used as landmarks to assist the location of the same FOVs over time. Tissue orientation over time remained stable in healthy kidneys (Supplementary Figure S2) and even enabled identification of identical tissue regions during strong pathological tissue remodeling as observed after ischemia-reperfusion-injury (Figure 5).

### 3.3 Validation of algorithms for Poisson-Gaussian noise removal in IVM data

The performance of denoising algorithms for noisy IVM imaging data was assessed quantitatively and qualitatively. Denoising and performance evaluation was done on a denoising validation dataset (Table 1), which was intentionally acquired with low illumination power and high detectors gains to corrupt weak fluorescence signals in the sample with high noise levels. Endothelial cells identified through endogenous tdTomato expression, were detectable in channel 1 and to a minor extent in channel 2. Renal tubular autofluorescence from flavoproteins and NADH, were detected in channels 2 and 3, respectively. Additionally, a strong second harmonic generation (SHG) signal from collagen fibrils in the renal capsule was detected in channel 3 (Supplementary Figure S1).

**FIGURE 2:**
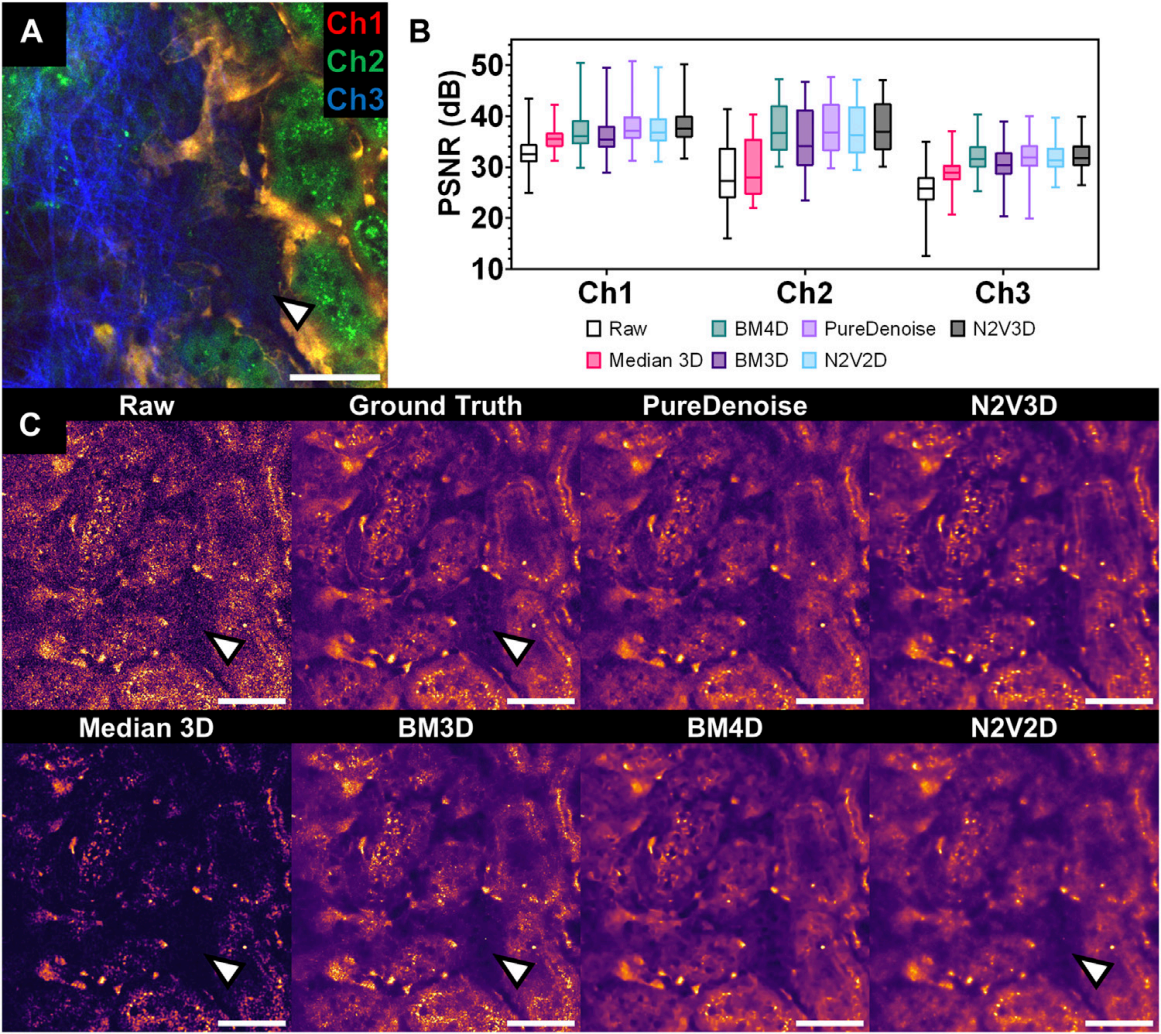
Performance of multiple denoising algorithms on an intravital 2-photon microscopy dataset corrupted by Poisson-Gaussian noise. **(A)** Composite intravital 2-photon microscopy image of from validation dataset (Table 1): Channel 1 (Ch1) shows endothelial cells expressing tdTomato (orange), channel 2 (Ch2) demonstrates renal tubular autofluorescence (green) and channel 3 (Ch3) depicts collagen fibrils in the capsule visualized through second harmonic generation (blue). Scale bar = 50 µm. **(B)** Quantitative assessment of denoising performance of multiple algorithms. Peak signal to noise ratio (PSNR) was measured for each slice and detection channel in the validation data set (n = 1,125 per channel) for raw data (Raw) and different denoising algorithms. Boxplot with median, 25th and 75th percentiles, and whiskers for minimum and maximum values. Note low PSNR levels for Median 3D filter in Ch2 and 3 (statistical comparison in Table 2). **(C)** Qualitative assessment of denoising performance of multiple algorithms. Side-by-side comparison of single channel (Ch2) intravital 2-photon images displaying the same field of view in raw data (raw), noise-free Ground Truth image (averaged from 25 noisy images of the same field of view), and after denoising with different denoising algorithms. Note poor recovery of dark fluorescent signals after denoising with Median 3D and 2D-based algorithms BM3D and N2V2D (arrowheads). Data is presented with a mpl-inferno lookup table depicting lower intensities in black and higher values are in light yellow. Scale bar = 50 µm.

PSNR measurements of raw data against GT stacks displayed the lowest levels for channel 3 (25.65 ± 3.81 dB), followed by channel 2 (28.73 ± 5.91 dB) and channel 1 (32.91 ± 3.20 dB). Denoising achieved improved PSNR measures for all tested algorithms (Table 2; Figure 2B). While the performance of BMxD, PureDenoise and N2VxD algorithms was largely comparable, we measured overall lower PSNR values after Median 3D filtering. Furthermore, algorithms exploiting 3D data from neighboring focal planes (PureDenoise, BM4D, N2V3D) achieved higher PSNR values than equivalent algorithms performing on single 2D images (BM3D, N2V2D), (Table 2).

**TABLE 2:** Image denoising performance on validation dataset (n = 1,125 slices) measured with mean PSNR and expressed in dB, highest values are highlighted with a bold font. The multiple comparisons were performed with Dunn’s test.

		Mean PSNR (dB)	Raw	Median3D	BM3D	BM4D	Pure denoise	N2V2D	N2V3D
**Raw**	Ch1	32.91 ± 3.20	—	****	****	****	****	****	****
	Ch2	28.73 ± 5.91	—	*	****	****	****	****	****
	Ch3	25.65 ± 3.81	—	****	****	****	****	****	****
**Median3D**	Ch1	35.6 ± 2.28	****	—	ns	****	****	****	****
	Ch2	29.8 ± 5.67	*	—	****	****	****	****	****
	Ch3	28.55 ± 3.59	****	—	****	****	****	****	****
**BM3D**	Ch1	36.48 ± 4.07	****	ns	—	****	****	****	****
	Ch2	35.42 ± 5.89	****	****	—	****	****	****	****
	Ch3	30.58 ± 3.44	****	****	—	****	****	****	****
**BM4D**	Ch1	37.3 ± 4.22	****	****	****	—	****	****	****
	Ch2	37.71 ± 4.68	****	****	****	—	ns	ns	ns
	Ch3	32.12 ± 3.00	****	****	****	—	ns	ns	ns
**Pure Denoise**	Ch1	38.25 ± 3.98	****	****	****	****	—	ns	ns
	Ch2	37.89 ± 4.91	****	****	****	ns	—	ns	ns
	Ch3	32.02 ± 3.48	****	****	****	ns	—	ns	ns
**N2V2D**	Ch1	37.92 ± 4.09	****	****	****	****	ns	—	****
	Ch2	37.36 ± 4.93	****	****	****	ns	ns	—	*
	Ch3	31.96 ± 2.82	****	****	****	ns	ns	—	ns
**N2V3D**	Ch1	**38.53 ± 3.93**	****	****	****	****	ns	****	—
	Ch2	**37.99 ± 4.83**	****	****	****	ns	ns	*	—
	Ch3	**32.36 ± 2.77**	****	****	****	ns	ns	ns	—

Qualitatively, all tested denoising algorithms achieved visual improvement when compared to raw data and recovered image signals while suppressing noise (Figure 2C). Denoising algorithms performed particularly well on channel 1, in which a rather bright signal from tdTomato-positive endothelial cells was recorded (Supplementary Figure S1). However, when restoring weak signals such as tubule autofluorescence recorded in channel 3, Median 3D filtering performed poorly, resulting in loss of signal when compared to GT data (Figure 2C). Similarly, the 2D algorithm BM3D did not fully restore very weak fluorescent signals (Figure 2C).

### 3.4 Automatic rigid image registration for sample drift compensation

To correct residual breathing and movement-induced image artefacts, we performed 2D registration using the SIFT image registration algorithm on Tseries or Zstacks.

**FIGURE 3:**
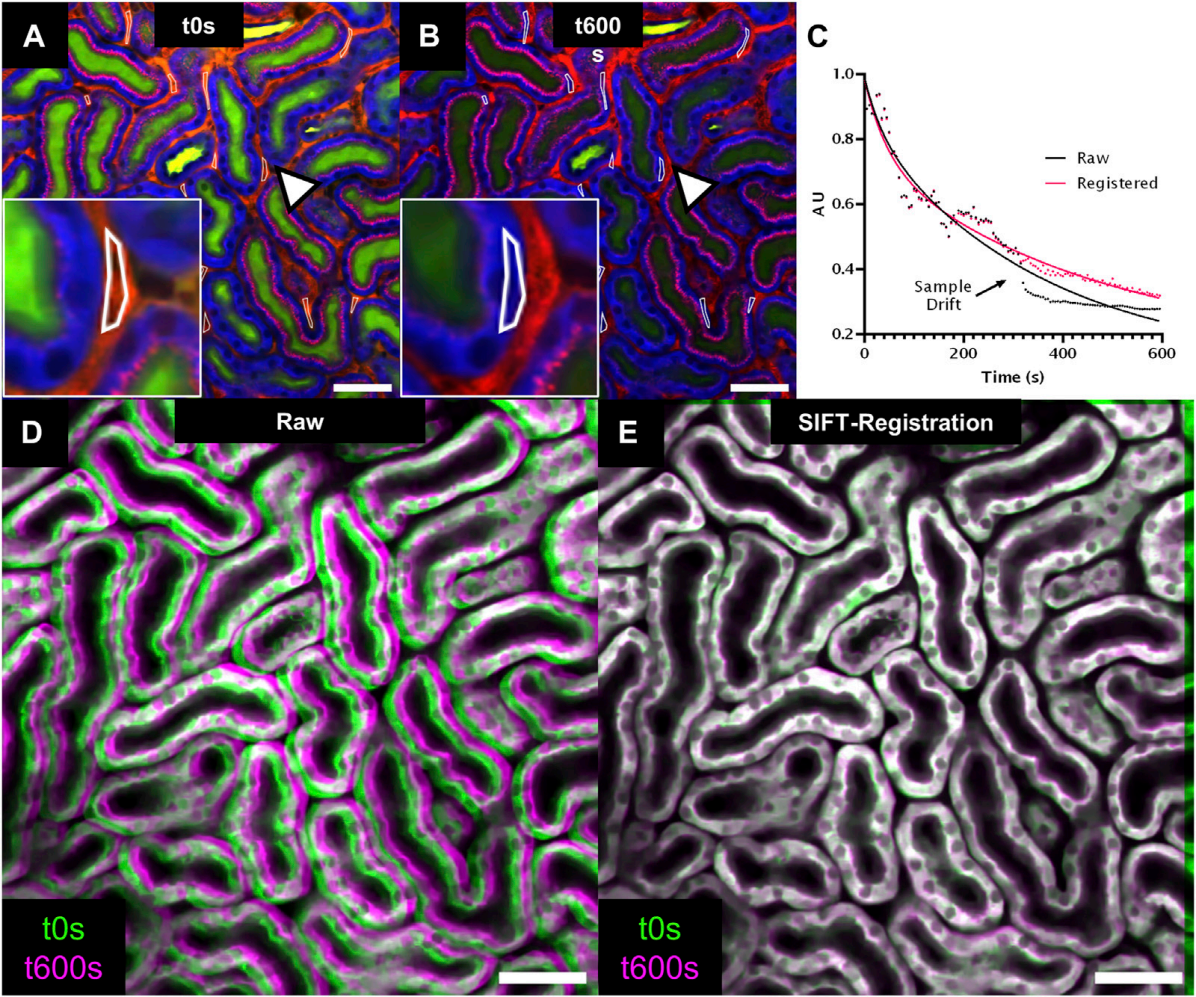
2D registration of time series (Tseries) with SIFT for bolus tracking experiment. **(A,B)** Intravital 2-photon microscopy images extracted from a Tseries acquired in a wildtype mouse kidney after injection of a FITC-Dextran 4kDa bolus injection (green). Images were extracted at time points t0s and t600s from the Tseries. Tubule epithelium is visualized in blue through tubule autofluorescence and peritubular vasculature and albumin uptake in proximal tubules is labeled in red through Alexa594-albumin (Table1). Multiple regions of interest (ROI, arrowhead, inset) are placed onto peritubular capillaries to assess vascular FITC-Dextran 4kDa fluorescence intensity over time. Note displacement of ROIs at t600s due to sample drift (arrowhead, inset, **(B)**. **(C)** Time-intensity plot (dotted line) and non-linear fitting (continuous line) of vascular FITC-Dextran 4kDa fluorescent decay assessed in raw (black) and SIFT 2D registered data (red). **(D,E):** Overlay of tubule autofluorescence signal from image time points t0s (green) and t600s (magenta) for raw **(D)** and SIFT 2D registered data **(E)** provides visual confirmation of sample drift compensation. Scale bars = 50 µm Temporal resolution = 5s.

2D registration was performed on a 600s-long Tseries recording of renal tubules after i. v. injection of FITC-Dextran 4KDa (Table 1). The raw data was affected by gradual image drifts beginning at t = 325s, which prevented accurate quantification of FITC-fluorescence decay in peritubular capillaries due to sudden displacement of measure ROIs induced by breathing movements (Figures 3A–C). Furthermore, an overlay of the first and last image frame of the Tseries demonstrated a clear sample drift (Figure 3D). Sample drift correction using SIFT revealed registration with sub-µm precision and aligned fine tubular and vascular structures with limited inter-frame shifts. Thus, SIFT image stabilization corrected displacement of measure ROIs and allowed accurate read-outs of FITC fluorescence signal intensity over time. Furthermore, the visual overlay of t0s and t600s frames from the registered stack confirmed successful drift correction (Figure 3E).

**FIGURE 4:**
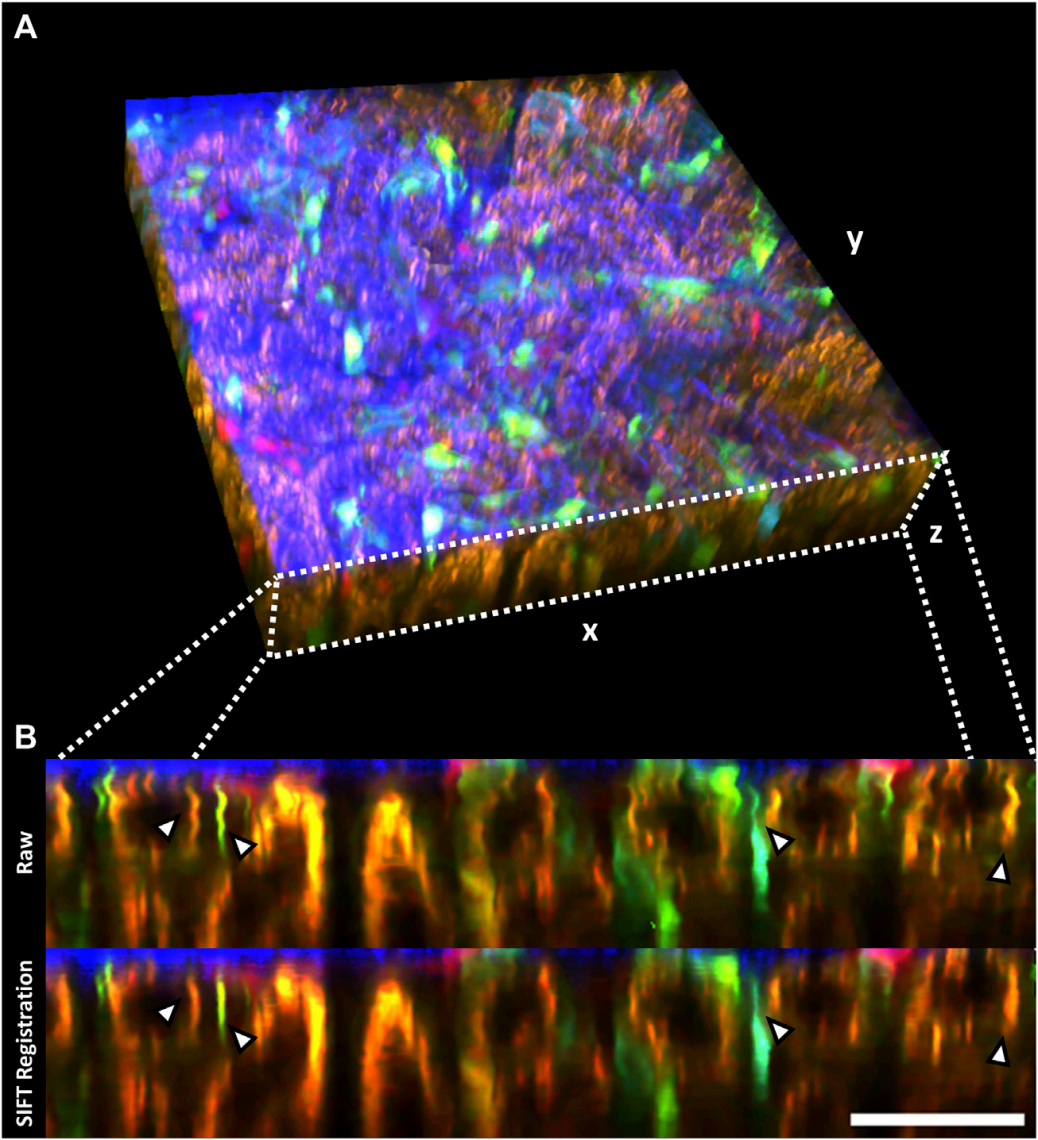
2D registration of volumetric data (Zstack) with SIFT minimizes sample drift-induced image artefacts. **(A)** Volumetric rendering of an intravital 2-photon microscopy Zstack acquired in a Cdh5-Confetti mouse. The renal capsule is visualized in blue through second harmonic signal. Endothelial cells expressing the Brainbow2.1 reporter system can be identified through 10 different color combinations (Table 1). **(B)** xz cross-section through the Zstack demonstrates image artefacts introduced by breathing movements in raw data (Raw, upper row). Thin fluorescently labeled endothelial cells show warped, wave-like appearance in the axial cross-section (arrowheads). SIFT 2D registration largely reduces sample drift on volumetric data (lower row, arrowheads). Scale bar = 50 µm.

2D registration of slices in volumetric stacks was performed on a Zstack recorded in the kidney of an anesthetized mouse encompassing a 295 × 295 × 55 µm tissue volume (Table 1), which demonstrated substantial breathing artefacts. Thus, XZ cross-sections identified endothelial cells and renal lysosomes of warped appearance due to inter-frame shifts (Figure 4B, upper row), which were successfully compensated through the SIFT algorithm in the registered stack (Figure 4B, lower row).

### 3.5 Manual landmark-based volumetric image registration

Analyzing longitudinal data holds the potential of comparing paired observations of tissue structure and function over time. To do so, corresponding tissue structures in serial imaging data need to be aligned, which can be achieved through 3D registration.

**FIGURE 5:**
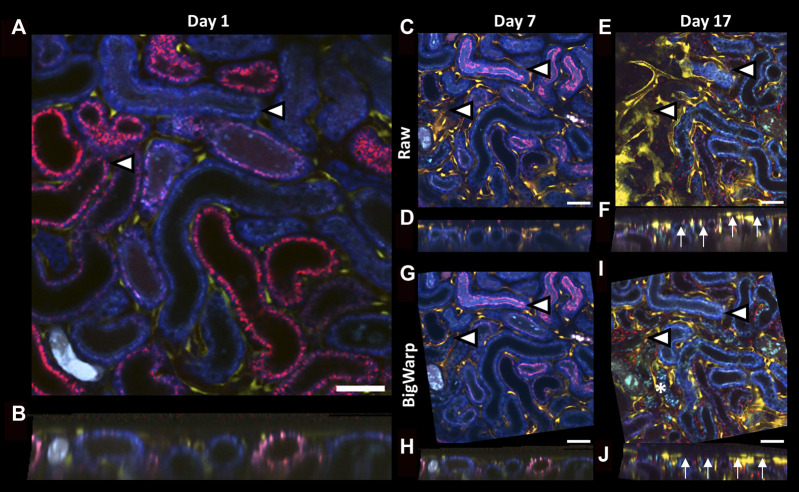
Landmark-based 3D registration of serial intravital microscopy data using BigWarp. **(A–J):** Representative 2D images **(A,C,E,G,I)** and xz cross sections **(B,D,F,H,J)** of volumetric serial intravital 2-photon microscopy data acquired in a Cdh5-tdTomato mouse kidney at day 1 **(A,B)**, 7 **(C,D,G,H)** and 17 **(E,F,I,J)** after ischemia reperfusion injury (IRI) before **(C–F)** and after **(G–J)** 3D registration using BigWarp. Arrowheads in 2D images indicate 2 representative tubules in serial data to demonstrate the effect of 3D registration and to reference equivalent locations in the field of view. Thus, severe tissue remodeling and tubule atrophy development at day 17 (I, asterisk) resulted in tissue shrinkage and changes in orientation that prevent simultaneous 2D displaying of tissue structures that used to be in the same plane (E, arrowheads). Note tissue shrinkage in day 17 xz cross section (F, arrows). In contrast, 3D registration using BigWarp successfully aligned tissue structures in serial imaging data (J, arrows). Scale bars = 50 µm.

To test manual landmark 3D registration with Big Warp, we used serial intravital imaging data collected from a mouse at days 1, 7 and 17 after ischemia-reperfusion-injury (IRI, Table1). Due to severe injury in the renal parenchyma and tubule atrophy, drastic tissue shrinkage in parts of the field of view could be observed at day 17 (Figure 5F). Thus, pronounced tissue remodeling prevented visualization of tissue structures that used to be in the same 2D plane (Figure 5E). Manual 3D registration using Big Warp (based on 15 landmarks in data from day 1, 7 and 17, respectively) not only corrected for slight z-rotations in serial data files but also compensated for tissue shrinking-mediated y-rotations and re-enabled the visualization of neighboring tissue structures in the same 2D plane for easy side-by-side comparison (Figures 5G, I). Day 17 cross-sections of raw unregistered (Figure 5F) and registered ZStacks (Figure 5J) demonstrate successful compensation of y-rotations.

## 4 Discussion

In this work, we describe a simple experimental setup and analysis pipeline for serial intravital imaging of the kidney, or other abdominal organs, using upright microscopes. We aimed to provide imaging and analysis tools that can be easily implemented by IVM practitioners with a biomedical background. The outlined protocols were developed and tested for laser scanning imaging systems with PMT/GaAsP detectors and may also be compatible to hybrid detectors and brightfield systems with camera-based detectors.

Abdominal organs are not anchored to any skeletal structure and subject to periodic breathing movements with mm-scale displacements in mice (Segars et al., 2004). This makes obtaining high resolution data challenging and demonstrates a major barrier when using upright microscopes, as uncompensated breathing movements prevent image acquisition of living abdominal organs. The presented custom 3D printed animal holder and wAIWs are key enablers to perform (serial) IVM of the kidney and other abdominal organs on upright microscopes. The setup proved easy to use and provided satisfactory tissue stabilization for IVM. To ensure full reproducibility, user-printable 3D model files were provided with this manuscript. A publicly available Tinkercad project will allow further customization of the holder to suit specific needs.

The first step in our image processing pipeline for serial IVM data is optional image denoising. We present multiple denoising algorithms, which all demonstrated major improvements of image quality when performed on a validation IVM dataset that was heavily corrupted by noise. Further, we provide detailed processing protocols to all different algorithms as well as multiple scripts for streamlined use in FIJI without requiring extensive experience in other programming environments such as Matlab.

Quantitative performance comparison of different denoising algorithms was based on PSNR metric to assess the numerical difference between signal in the ground truth and in the raw/denoised image, respectively (Lin and Kuo, 2011; Laine et al., 2021). In addition, we performed qualitative evaluations of denoising performance. While determination of the qualitatively best algorithm remains a matter of subjective evaluation, our data indicate that all tested denoising algorithms performed comparably well, while commonly used Median 3D filtering failed to accurately restore weak image signals. Which denoising algorithm to use largely depends on practical choices based on computational constraints or the nature of the data. Furthermore, software availability might be a consideration, as BMxD algorithms are supported by the commercial product, Matlab. BMxD and PureDenoise algorithms do not require training and can be readily applied on IVM datasets. Nevertheless, they can be computationally costly with long runtimes and high memory consumption. In contrast, N2V neural networks require training, which may not be practical on small data sets or when appropriate GPU acceleration is not available. However, trained neural networks tend to be computationally cheaper at prediction time, especially when run on GPUs. Therefore, their application may prove extremely advantageous on large datasets where denoising with traditional algorithms may incur in long computation times.

IVM techniques can collect raw data of high visual clarity (Schiessl et al., 2018; Desposito et al., 2021). However, certain experimental questions or conditions may demand fast scanning speed and low illumination power to preserve sample viability (Laine et al., 2021). Similarly, acquisition of weak signals might demand increased detector gain. Such imaging conditions often result in noisy images. Here we show that image denoising can be very beneficial to restore signal in noisy raw data. However, denoising algorithms often rely on statistical models of noise, non-linear numerical transformations, and other assumptions which may fail to faithfully model the physical processes emerging as image noise (Laine et al., 2021). Thus, denoising algorithms may introduce artefacts and caution should be applied when using denoised images for quantifying pixel intensities or studying the morphology of subtle biological structures (Meiniel et al., 2018; Kay, 2022). On balance, image denoising can support IVM applications and there is sound technical and scientific literature supporting its validity. Our manuscript provides easily accessible denoising pipelines that will assist IVM data processing.

Passive mechanical imaging stabilization, as demonstrated in this manuscript, can decrease breathing and movement artefacts to a significant extent but will not completely prevent them (Vinegoni et al., 2014). In contrast, active motion compensation/stabilization systems are capable of detecting and/or avoiding sample drift that can be advantageous for IVM (Vinegoni et al., 2014; Soulet et al., 2020). However, these applications are far less common and demand advanced equipment and expertise for their implementation. Furthermore, they generally require lower acquisition rates and/or higher data storage as, for example, the scanning process must be gated (i.e., paused) to breathing movements (Dunn et al., 2014; Vinegoni et al., 2014; Soulet et al., 2020).

As an easier solution to remove remaining movement artefacts, we demonstrate the use of 2D rigid registration using the SIFT algorithm (Lowe, 2004) and a translational transformation with FIJI. Previously, we also applied the “Descriptor-based series registration” algorithm (Preibisch et al., 2010) to stabilize IVM data (Engbjerg et al., 2022; Grimm et al., 2022). However, we found that SIFT was generally easier to tune for good performance and computationally less expensive. SIFT performance for 2D image registration depends on detectable image features. As the algorithm performs on a single channel basis, volume registration may be challenged by inhomogeneous signal distributions across total volume and individual detection channels. To overcome this limitation, our SIFT registration pipeline involves generating a “registration channel” through mathematical signal addition of all detection channels in the respective data set. This approach maximized detectable image features in the data. While manual performance of all required steps for this processing would take considerable time, we provide a ready-to use script, which performs all required steps automatically to run in FIJI and further allows batch processing large datasets.

Longitudinal datasets are key to understanding how biological structures evolve and function over time (Cunha et al., 2014). Correlating longitudinal data often requires volumetric registration to compensate for differences in tissue or organ orientation over time and are typically facilitated by human-placed landmark approaches, automated algorithms or a mix of both. In this work, we demonstrated landmark-based volumetric registration of serial IVM data using the FIJI plugin BigWarp (Bogovic, 2016). Thus, human-placed landmarks identify structural correspondences between a reference and a moving volume and facilitate alignment of the two volumes. BigWarp requires a small number of landmarks to provide good alignments with a rigid rotation transformation and is computationally cheap. Furthermore, it requires limited user training to become proficient. Affine or non-rigid locally deformable transformations are potentially useful to register data acquired with different resolutions (e.g., aligning an AtlasScan to a higher resolution stack) and have been exploited for correlative light-electron microscopy (CLEM) (Karreman et al., 2016; Walter et al., 2020). However, a major concern of non-rigid transformations for longitudinal studies is that it can match the size or shape of image structures in the moving stack to the reference stack. Thus, non-rigid transformations may hide or manipulate local changes in size or shape of serially recorded biological structures that are truthful (Csapo et al., 2012), as, for example, during injury-induced tissue remodeling (Figure 5). Thus, we purposefully chose rigid over non-rigid transformation to preserve local tissue alternations and used 2D SIFT registration to compensate for breathing-induced sample drift.

Landmark-based registration requires human operators, which can be limiting when working with large datasets. As a result, automated volumetric registration algorithms have been extensively explored in medical imaging practice (Viergever et al., 2016; Keszei et al., 2017). Medical image registration is an active research field. However, preclinical application of automated registration algorithms is difficult due to the absence of broadly applicable protocols and customized parameter presets that were extensively validated for specific IVM applications. Furthermore, there is limited overlap between software tools and file formats used by bioimaging and medical communities. Nevertheless, increasing applications of lightsheet microscopy or longitudinal IVM, demand for new tools and interfaces to volume registration. Indeed, new deep learning approaches have shown promising results and may remove the need for extensive parameter optimization (Balakrishnan et al., 2019; Haskins et al., 2020). Furthermore, initiatives such as FIJI Elastix-Wrapper (Tischer, 2019) for the Elastix medical registration toolset (Klein et al., 2010; Shamonin et al., 2014), or BigDataViewer (Pietzsch et al., 2015) to visualize TB-scale datasets, are promising developments.

Serial IVM of abdominal organs such as the kidney holds a huge potential to understand (patho-) physiological tissue dynamics. However, IVM data acquisition and processing not only requires expensive equipment but also significant expertise. This work aimed to provide tools to lower the barrier of entry to these exciting techniques to make IVM of the kidney and other abdominal organs more accessible to biomedical scientists. The provided animal holder was designed to be reproduced and the presented software scripts demonstrate easy to use pipelines for longitudinal visualization and analysis of IVM bioimaging data. We hope that this work will facilitate exciting new developments.

## Data Availability

An editable version of the animal holder is available on Tinkercad’s website at https://www.tinkercad.com/things/cPe673DgPlL and future iterations of the software scripts will be hosted on Github at https://github.com/DSbioimaging/IVM-Processing-Toolbox.

## Supplementary material

Supplementary Figure S1 Performance of multiple denoising algorithms on individual detection channels of the validation dataset. **(A–C)**: Representative intravital 2 photon microscopy collages showing raw data (Raw, top left), the N2V3D-denoised image (top right) and respective ground truth image (GT, bottom) for detection channel 1 (Ch1, A), channel 2 (Ch2, B) and channel 3 (Ch3, C), of the denoising dataset (Table 1). **(D–F)**: Magnifications of respective insets in A–C as raw data (Raw), GT and after Median 3D or N2V3D processing for Ch1 **(D)**, Ch2 **(E)** and Ch3 **(F)**, respectively. Note failure of weak signal restoration after Median 3D filtering (arrowheads). Data is presented with a mpl-inferno lookup table depicting lower intensities in black and higher values are in light yellow. Scale bars = 50 µm.

Supplementary Figure S2 Landmark-based 3D registration of serial intravital microscopy data using BigWarp. **(A)**: Representative 2D images of volumetric serial intravital 2-photon microscopy data acquired in a control Cdh5-Confetti mouse kidney at day 1, and day 7 with and without 3D registration with BigWarp. Endothelial cells expressing the Brainbow2.1 reporter system can be identified through 10 different color combinations (Table 1). Note relatively stable tissue orientation over time. Small misalignments between day 1 and day 7 are compensated for through 3D registration (arrowheads). Scale bars = 50 µm. **(B)**: Principle of BigWarp 3D registration. Manual landmark placing identifies common tissue features between a reference and a moving 3D stack acquired at different time points.
