# Supplementary material for: Serial intravital 2-photon microscopy and analysis of the kidney using upright microscopes
Source: Front Physiol. 2023 Apr 24;14:1176409. doi: 10.3389/fphys.2023.1176409 (PMC10164931; doi:10.3389/fphys.2023.1176409)
Supplement: Supplementary file 1 [file DataSheet2.PDF]

# *Landmark-Based Registration of Zstacks for Serial IVM of the Kidney*

## 1 Installation

- BigWarp is part of FIJI, download at www.fiji.sc
- Documentation at https://imagej.net/plugins/bigwarp details every function of the plugin.

## 2 First steps and setup

- Open two ZStacks acquired at two different time points.

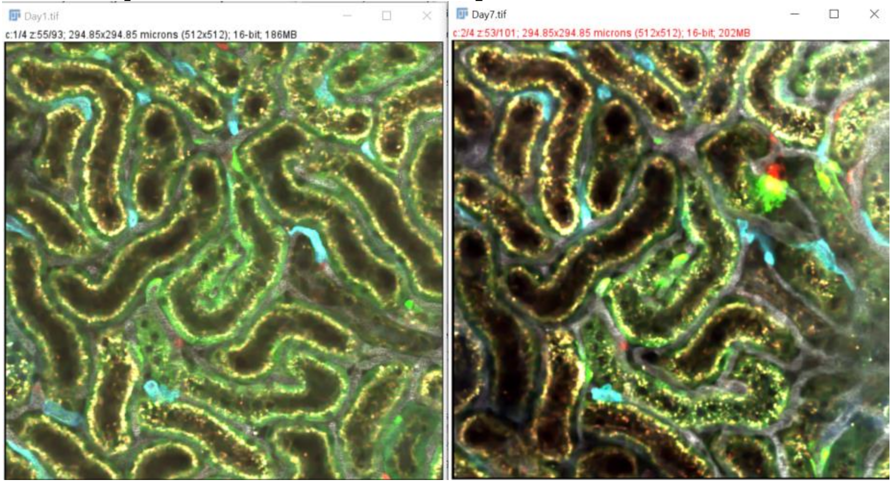


- Open BigWarp by clicking on Plugins→BigDataViewer→BigWarp

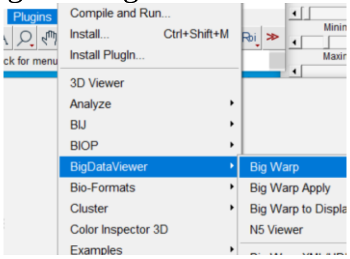


- Set the target (reference) stack and the moving stack. E.g. day 7 is the moving stack while day 1 is the target.

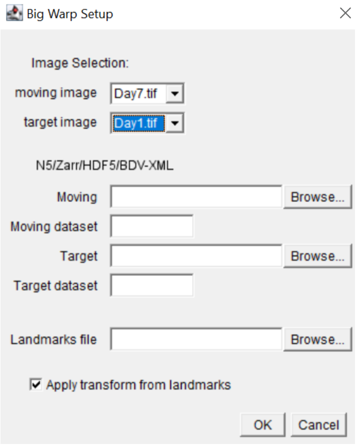

- The BigDataViewer will open: the target stack, the moving stack and a “Landmarks” window.

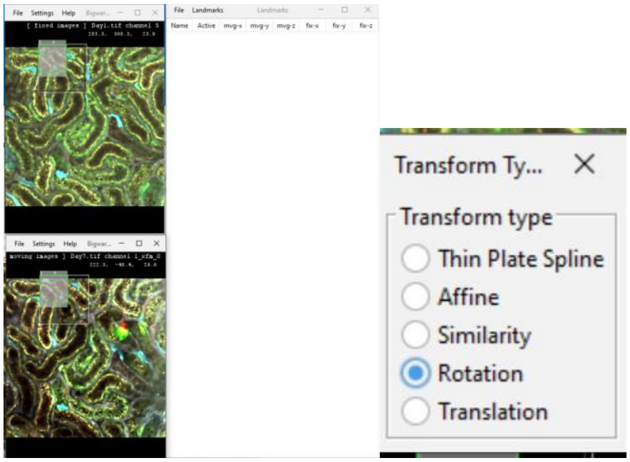


- Press “F2” to set the Transformation to “Rotation” then close the “Transform Type” Window.
- For added comfort adjust the window size then the contrast of each channel by hovering the mouse on the right side of a stack window. A blue arrow will appear, click on it to open a side panel where the contrast of each channel can be adjusted and the color of each LUT changed. In the figure below, channel 1 (White grayscale LUT) was disabled in both stacks by moving the contrast slider for the minimum value to the rightmost position.

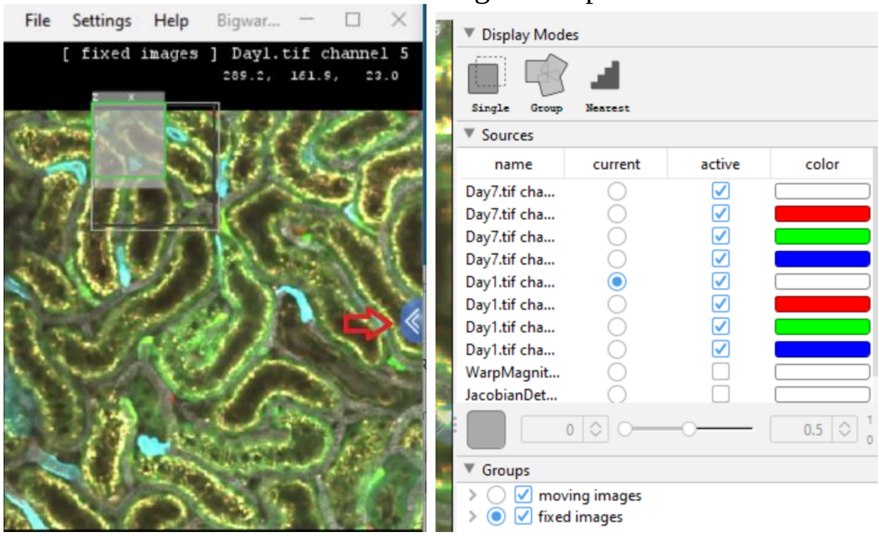


- BigWarp is operated by a combination of key bindings and mouse inputs. These are the basic commands (read the documentation for more):
  - **UP and Down arrows**, adjust the zoom
  - **Mouse wheel,** adjust the z plane in current viewer window
  - **X,Y, and Z**, set the axis of rotation along one of these dimensions
  - **Left and Right Arrows**, rotate around the selected axis
  - **Left mouse click and drag**, free hand rotation around selected axis
  - **Right mouse prolonged click and drag**, XY translation of the current view
  - **R**, reset current viewer
  - **Spacebar**, enter or exit Landmark mode
  - **T**, apply transformation according to current landmarks (minimum of 4)
  - **Q**, align the non-active viewer window to the active viewer window.

## 2.1 Landmark Registration

- Scroll with the mouse wheel and rotate the moving image to find a rough initial alignment of the stack and to visualize common anatomical landmarks.
- Locate corresponding structures in the two stacks by changing the z plane with the mouse wheel.

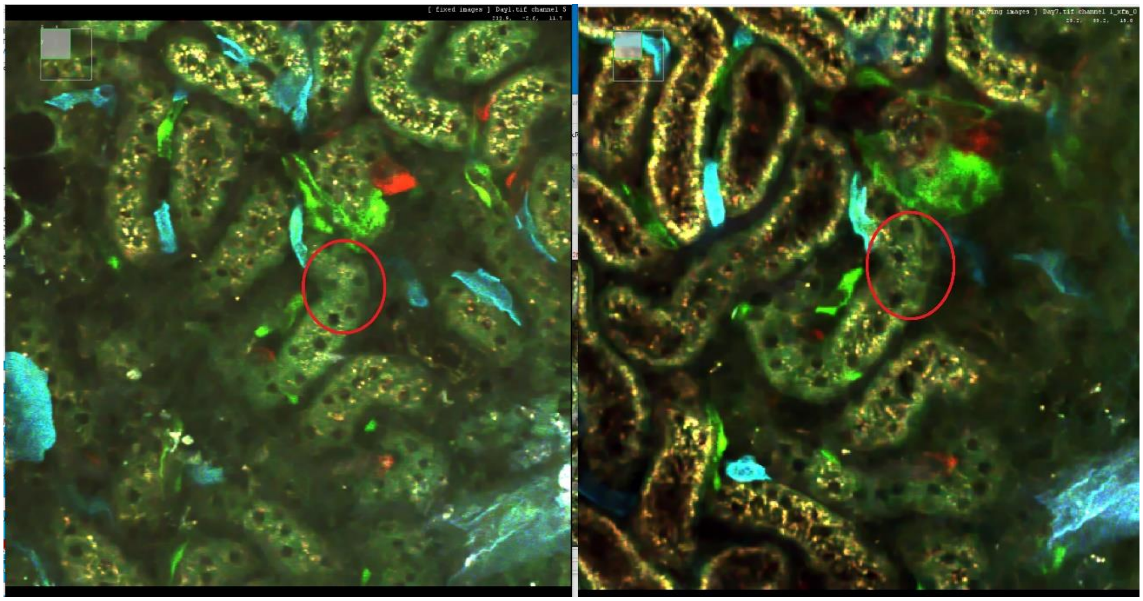

- Press “Spacebar” to enter the landmark placing mode.
- Click in the moving window on a landmark and then click on the corresponding feature of the target image. A landmark indicator will appear.

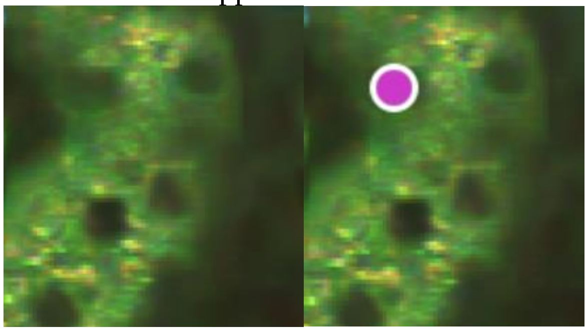

- The landmark mode can be disabled by pressing “Spacebar” again, this will return BigWarp to Navigation mode.
- Landmarks can be edited and deleted in the “Landmarks” window.
- Place the first 4 landmarks then press “T”, select the fixed window and then press "Q”. The moving image should now appear transformed and approximately aligned to the fixed stack view.

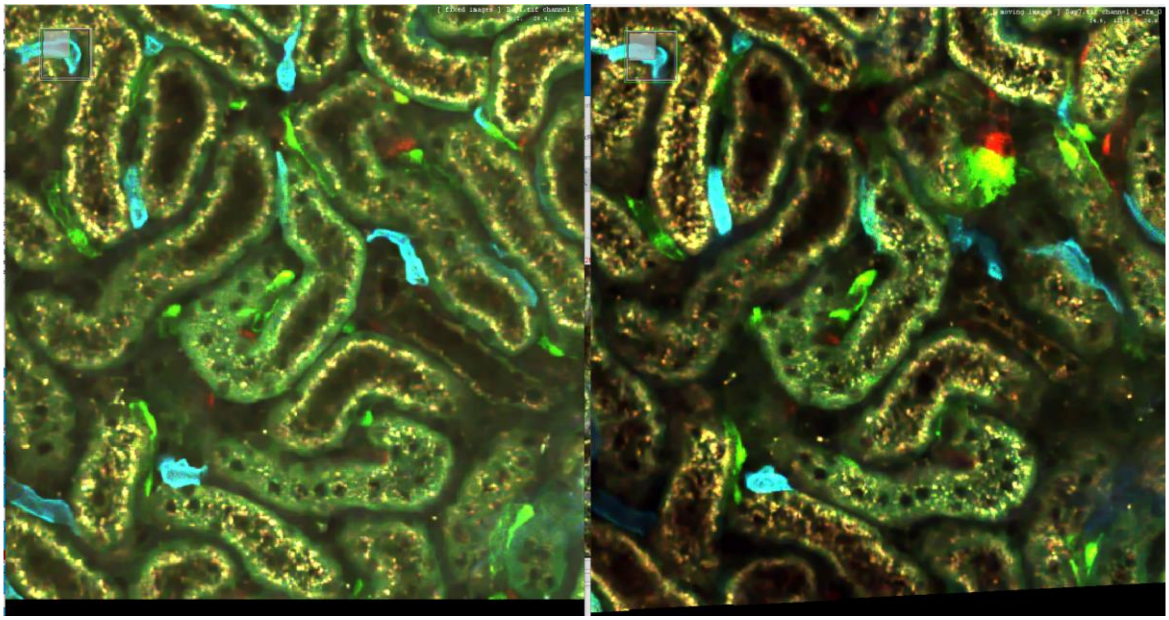

- Continue adding more landmarks until the alignment is satisfactory. Then save the landmarks to a .csv file for future use. In the “Landmarks” window click on File→Export Landmarks.

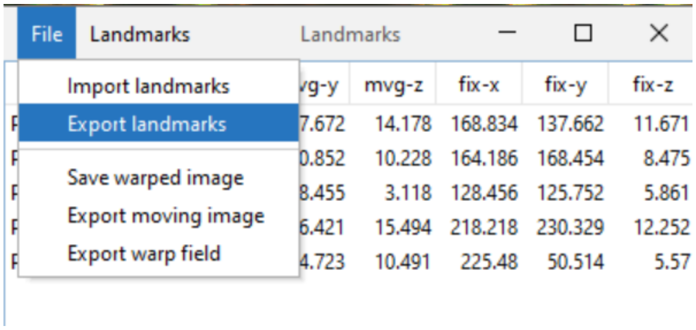


## 2.2 Exporting the Registered Moving Image

- In the “Landmark” window click on File→Export moving image
- The “Apply Big Warp transform” window will open. Default settings should be adequate for most applications, so click on “OK”. Big Warp will apply the transformation and a new registered stack will appear. The black region in the registered stack is caused by the rotation by BigWarp to align it to the reference stack.

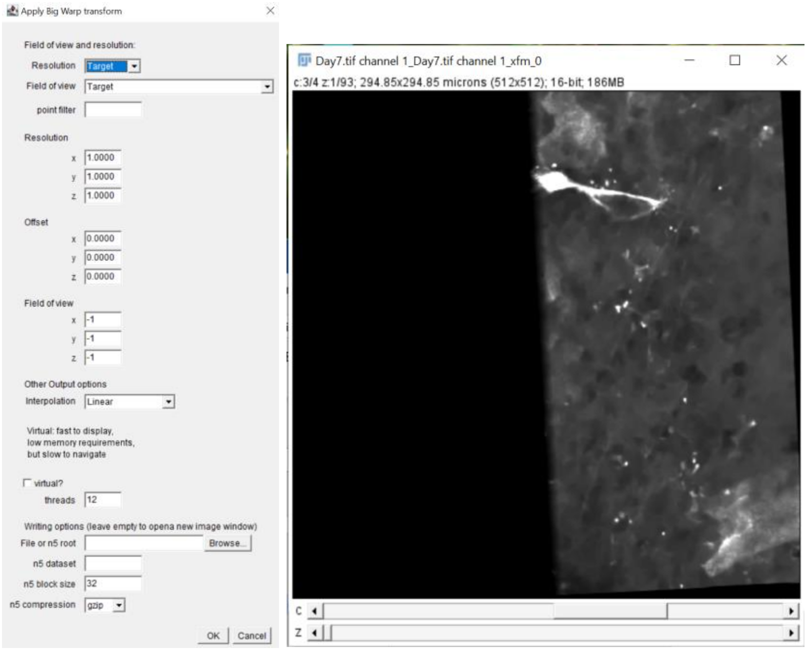


- The channels of the registered stack are visualized in the Default ImageJ modality as separate; it may be preferable to have a composite view. Click on Image→Colors→make composite.

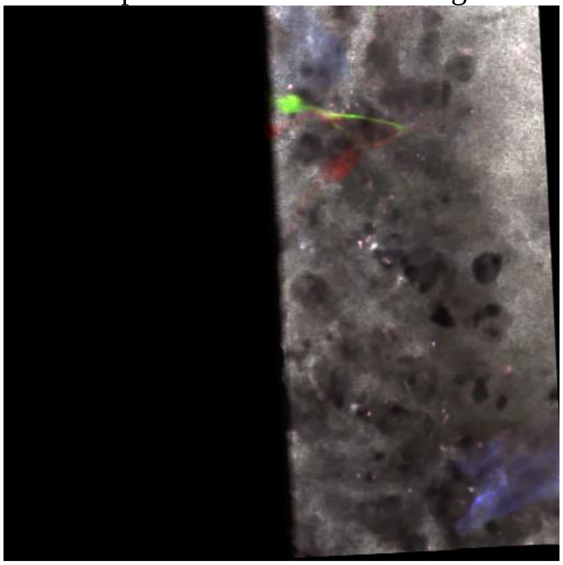

- The file is now ready to be saved.
- To obtain a 4D stack, it is possible to concatenate the first timepoint with stacks acquired at later time points using the Concatenate command at Image→Stacks→Tools→Concatenate. Enable the option "Open as 4D image" and click "OK"

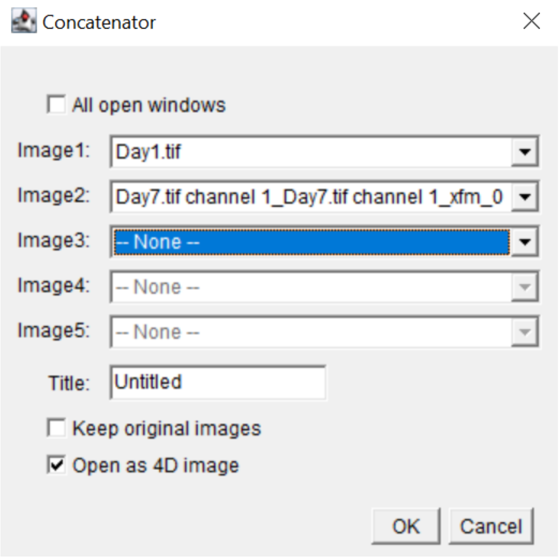


- BigWarp can now be safely closed to perform further registrations.
- 10-15 landmarks are generally sufficient for rigid registration.
