# Supplementary material for: Serial intravital 2-photon microscopy and analysis of the kidney using upright microscopes
Source: Front Physiol. 2023 Apr 24;14:1176409. doi: 10.3389/fphys.2023.1176409 (PMC10164931; doi:10.3389/fphys.2023.1176409)
Supplement: Supplementary file 2 [file DataSheet3.ZIP › BigStitcher-DenoisingValidation.pdf]

Protocol for Performance Validation of Image Denoising Algorithms for Intravital Microscopy Data

1 DATA ACQUISITION FOR DENOISING VALIDATION

- The protocol assumes that denoising algorithms will be tested on a 4D stack acquired with intravital microscopy, it is largely applicable to simpler use cases with some minor modifications (e.g. the use of simple drift correction to prepare a ground truth frame)
- Acquire a 4D stack (xyz+t) with at least 25 frames, more frames should result in a smoother ground truth. Imaging settings should be similar to the ones used for the raw data that will be denoised.
- Denoising algorithms are validated against a ground truth obtained by averaging the time points of the 4D stack. Averaging is done on a pixelwise basis and sample drift may result in a blurry ground truth image since equivalent structures would be in different locations over time. For this reason, it is necessary to register the data before averaging for the ground truth. The same registration transforms must be then applied to all the denoised and noisy images.

2 RAW DATA PREPARATION

- Open the 4D stack in FIJI
- In the workflow it is assumed that the 4D stack will be processed with one of the provided denoising scripts which have been implanted to perform batch denoising on single 3D stacks. Therefore, each timepoint must be split to a separate file.
- To split the time points use the provided the macro "Split_Timepoints" for this purpose. Drag the macro file on the main FIJI window and press run in the macro editor window then choose a folder where to save the single stacks.
- 3D Sample correction in the following steps can be attempted on the single noisy stacks but it may fail due to noise. To avoid this problem, denoised split 3D stacks may be used to calculate the transformations necessary to align all the time points. Please refer to the relevant protocol for using the denoising scripts.
- In this protocol BM4D-denoised stacks were used for registration and drift correction. The computed transformations were then applied to the noisy 3D split stacks.

3 REGISTRATION OF TIME POINTS IN BIGSTITCHER

- BigStitcher must be installed as a FIJI plugin from its update site. The documentation at https://imagej.net/plugins/bigstitcher/index explains the process and provides extensive guidance on all the many features of the software.

3.1 Creation of BigStitcher Dataset and data import:

- Open FIJI and add the "Big Stitcher" update site then update and restart the program.

- Open BigStitcher (Plugins→BigStitcher→BigStitcher) and create a new dataset with all time points in the same folder as the split 3D stacks. **This is important when applying the computed transformations to other 4D stacks.**

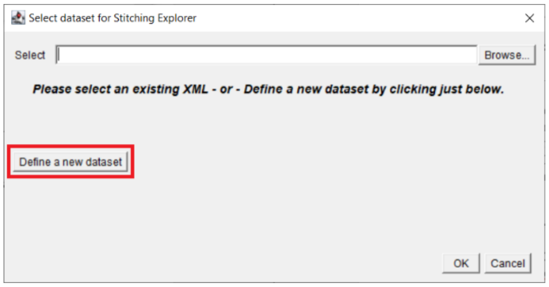

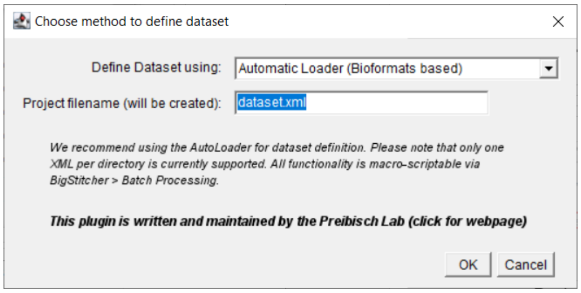

- Select the files to register in the next windows, the denoised folder should be empty aside from the clean stacks but wildcards and file size can be used to filter undesired data.

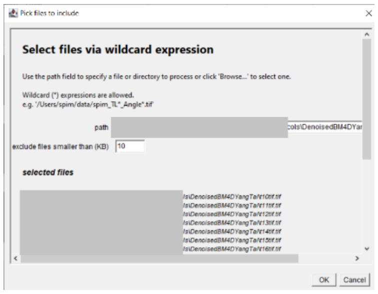

- BigSticher was originally developed for lightsheet microscopy where data can contain multiple views of the sample. For this reason, it is necessary to define the metadata in the file, set the Bioformats channels as channels and the numerical pattern for the different files (t1, t2, t3, t…) as time points.

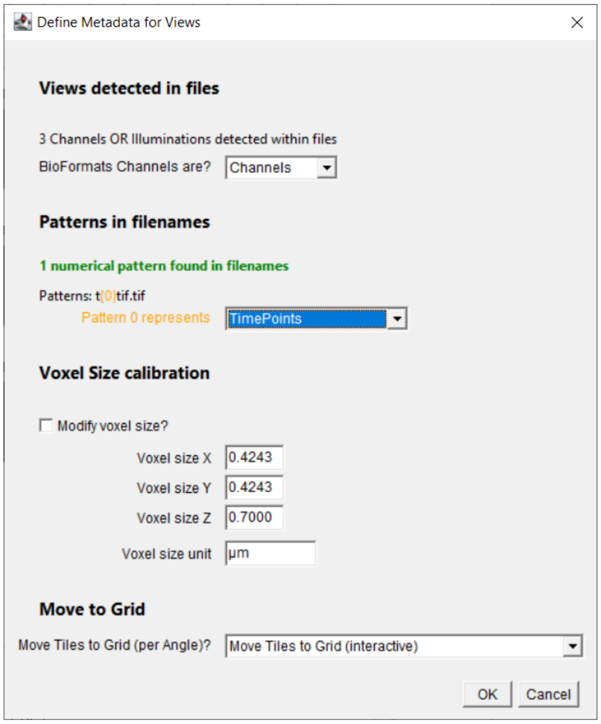

- Now define whether the BigStitcher dataset will be loaded from raw data files or from a newly created HDF5 then click OK.

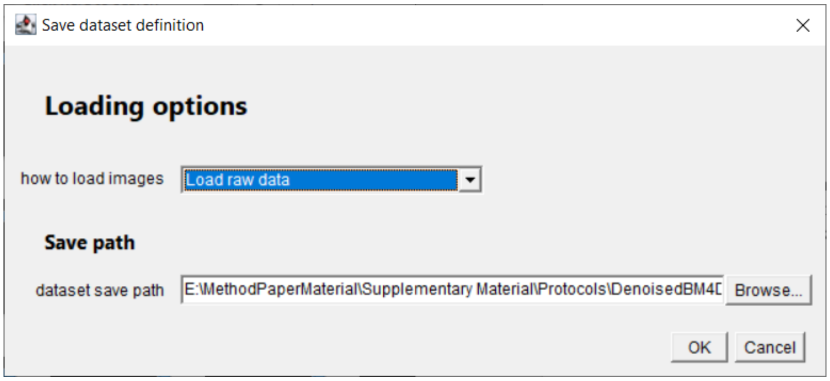

- In the Multiview Explorer window select all channels and time points

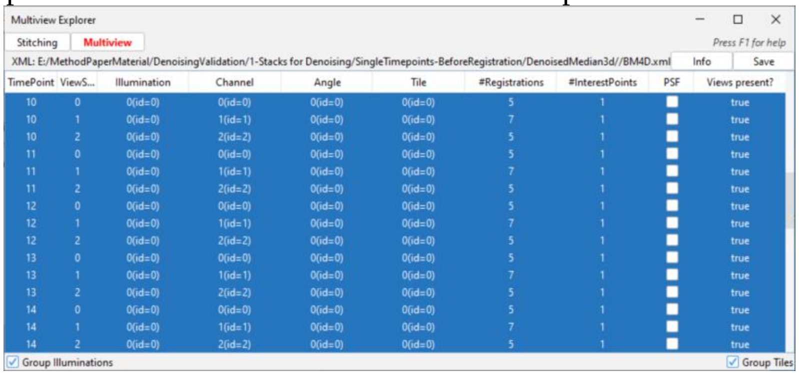

- It is now time to detect some tissue features in all channels for registration. Right-click over the channels in the multiview explorer window and under Processing→Detect Interest points

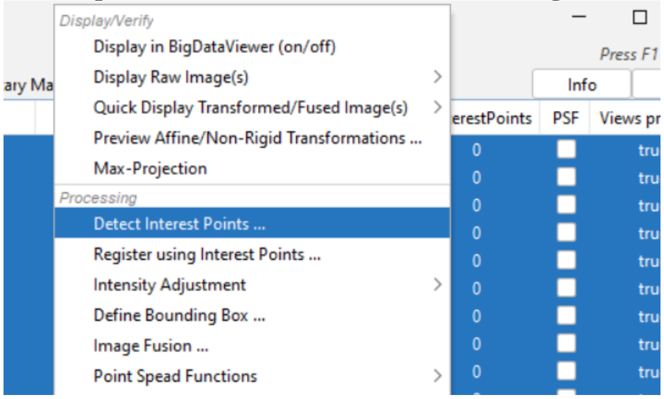

- Use the default Difference of Gaussian features and leave the rest to default, “Limit amount of detections” can be used for shorter runtimes Leave the Difference of gaussian (DoG) options to default.

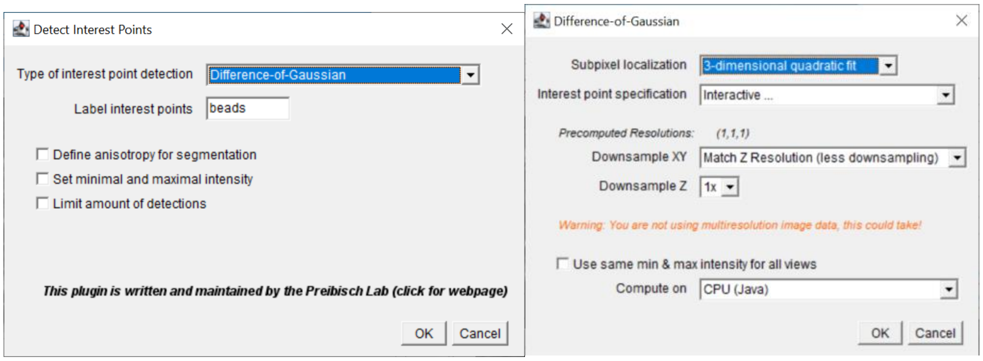

- Next choose a channel to use to interactively adjust the DoG settings.

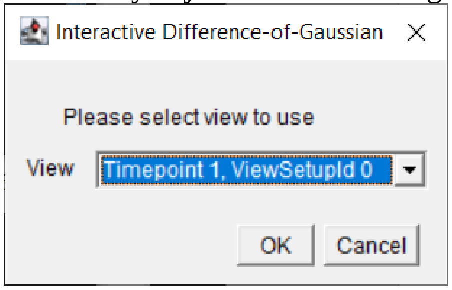

- Two new windows should now open, one with a browsable stack from the selected channel, the other contains two sliders that allow to adjust the DoG sigmas and the intensity threshold. The effect of the settings can be seen in the stack window. In the manuscript the sigma was set to 7.0075 and the threshold to 2.4468466E-4. These settings must be empirically adjusted for different datasets. A smaller sigma generally leads to more detections and the threshold should be adjusted to be above the background. Too many detections may lead to registration failure and long computation times, but too big of a sigma may lead to registration failure because of too few features not representing the general structure of the sample.

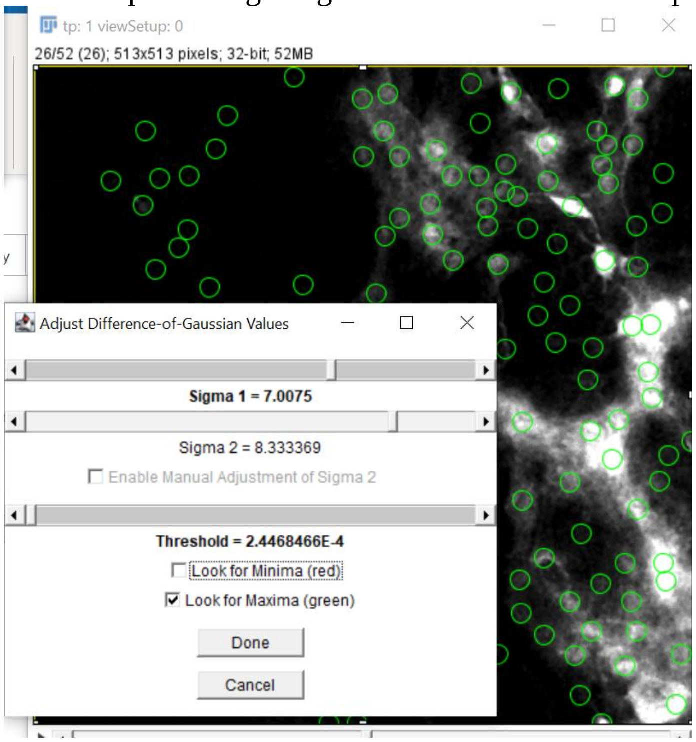

3.2 First Registration Pass:

- The goal of the first registration pass is to provide a rough alignment within each timepoint and over time. This is accomplished with a Translation model (translation along the xyz axes).
- Right click over the channels in the Multiview explorer window and under Processing→Register using Interest Point
- In the "Basic Registration Parameters" window select the "Fast descriptor-based (rotation invariant)" algorithm, "All-to-all time points matching with range" and enable "Group channels"

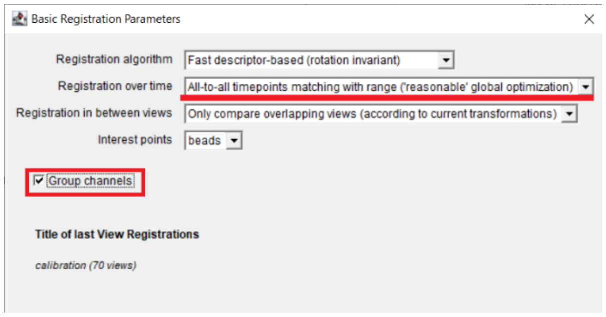

- Next select "Map back to first view using translation model" if you want to move all the time points relatively to the first one. Choose a "Translation" model.

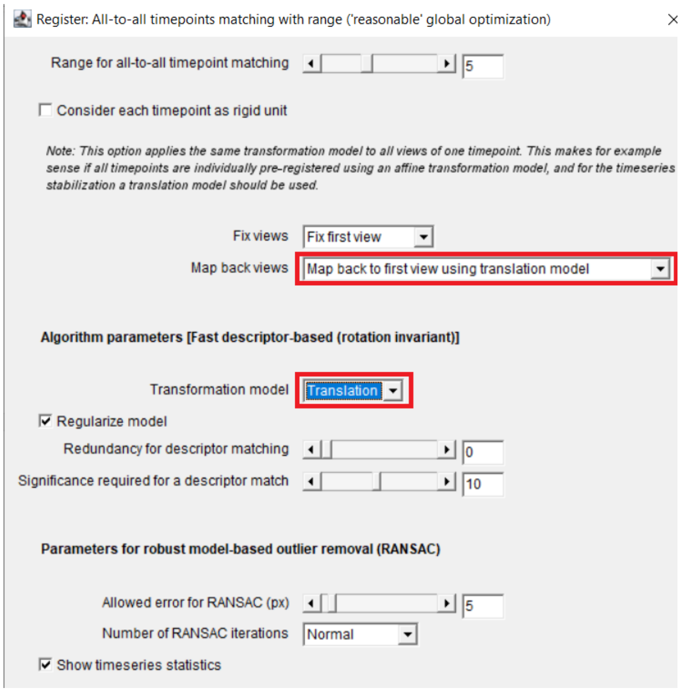

- In the "Regularization Parameters" window choose "Identity" and leave everything to default in the "Interest point grouping" window. Click OK and wait for the registration to run.

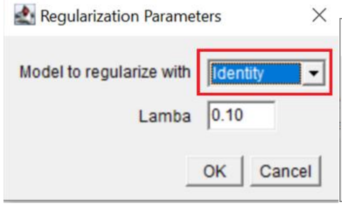

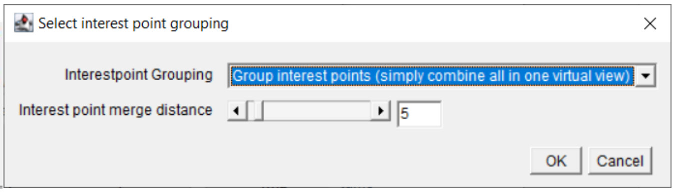

- A new window to evaluate the registration quality will now appear. If the correspondence ratio is too low, it means that the registration failed. The process can be attempted with different DoG settings until a good registration is obtained.

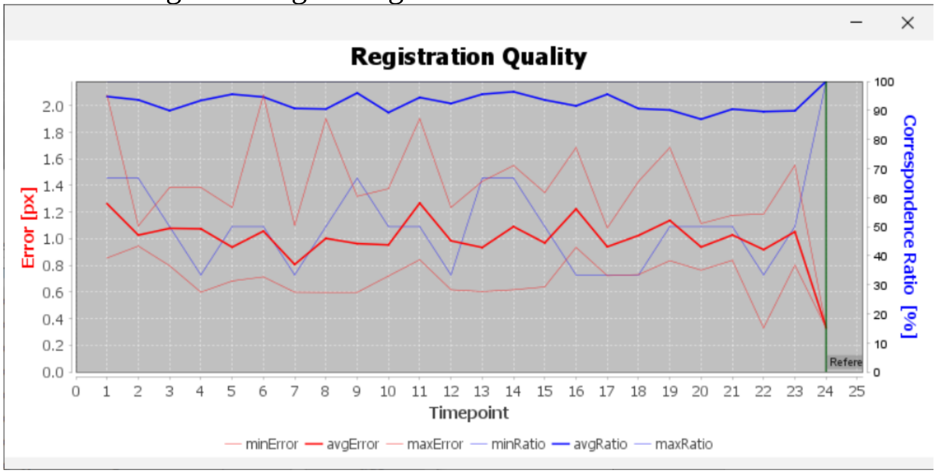

3.3 Second Registration Pass

- The second pass refines the results of the first pass with a different registration algorithm.
- Begin a second registration by right-clicking over the selected channels in the multiview explorer window and selecting Processing→Register using Interest Point
- Now select “Assign closest-points with ICP (no invariance)” and enable “Group channels”

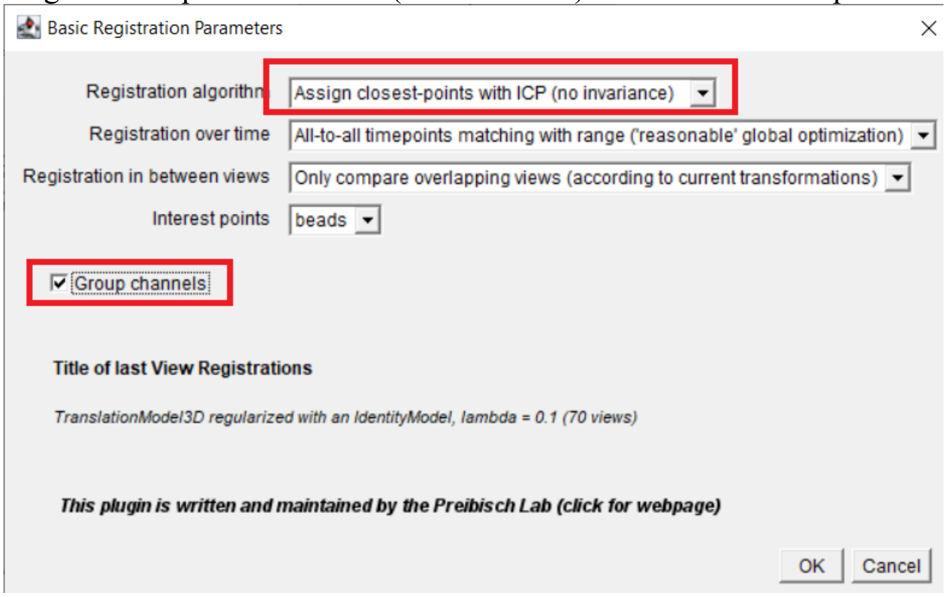

- In the next window select the “Translation” in the Transformation model dropdown menu, leave everything on default.

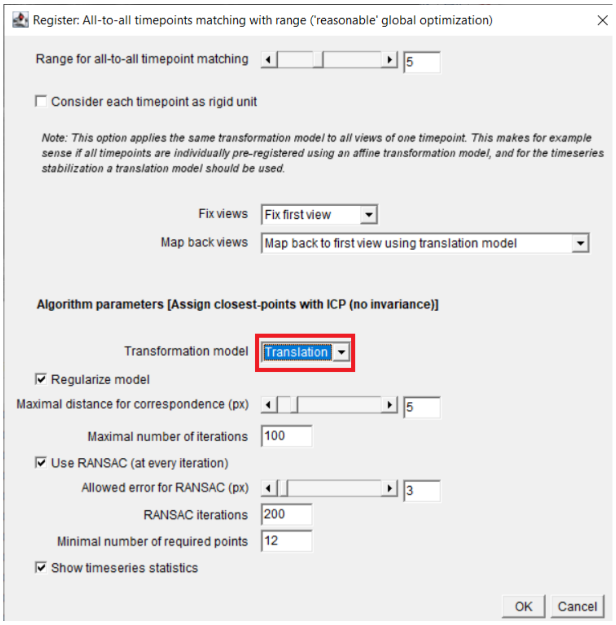

- Choose “Identity” in the “Regularization Parameters” window. Leave every setting on default in the select interest point grouping options.

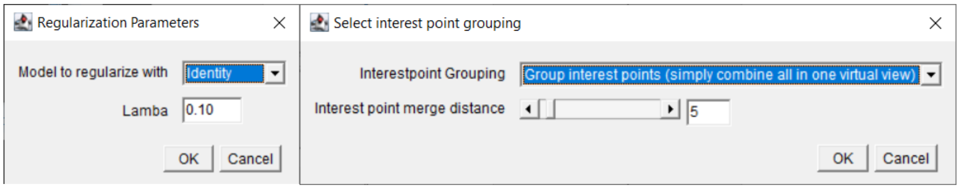

- The registration algorithm will now run and refine the alignment. This can be verified by the Correspondence ratio in the Registration quality plot. In this case the ratio plot is barely visible due to the value is 100% throughout the stacks (blue arrows).

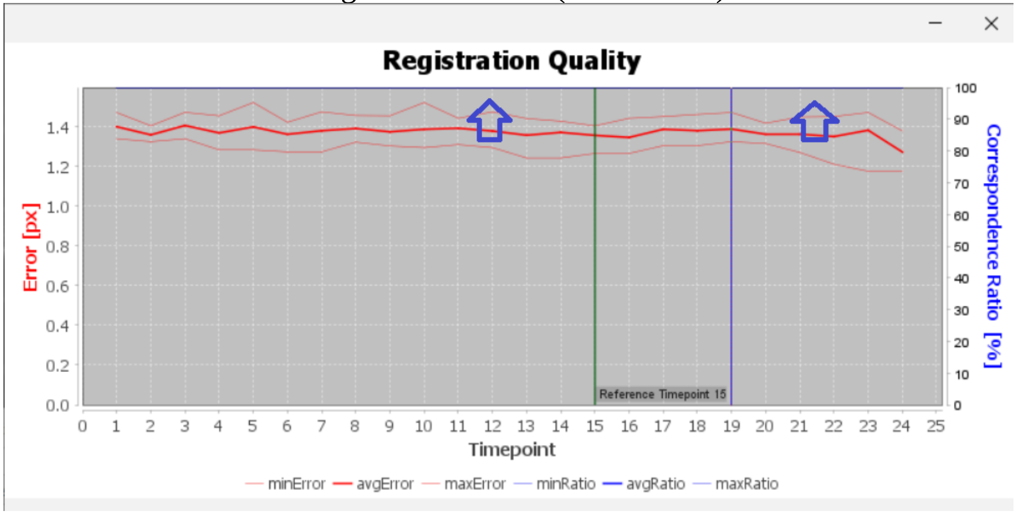

3.4 Saving the Computed Transformations and BigStitcher Dataset

- The transformations to align the single 3D stacks for each timepoint are now computed and must be saved in a BigStitcher project file to be applied on data processed with other

algorithms and/or the raw noisy data. In the Multiview Explorer window click on the Save button. This will save the points used for registration and the transforms to the originally chosen folder in section 3.1.

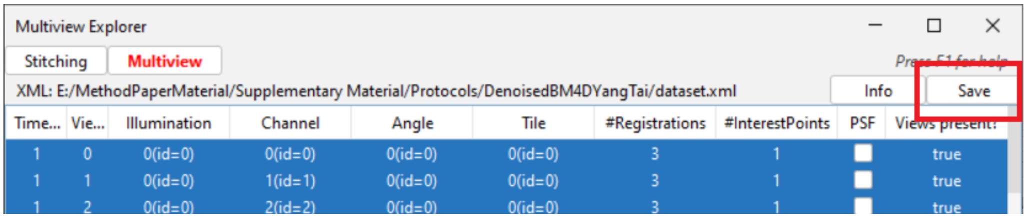

3.5 Export Registered Channels:

- Right click over the channels in the multiview explorer window and under Processing→ImageFusion
- In the "Image Fusion" window select "All views", "16 bits" pixel type (or lower if the raw data has a lower bit depth), enable "Preserve original data anisotropy" if data was scanned with anisotropic voxel size, produce one fused image for each timepoint and channel and save as compressed TIFF stacks.

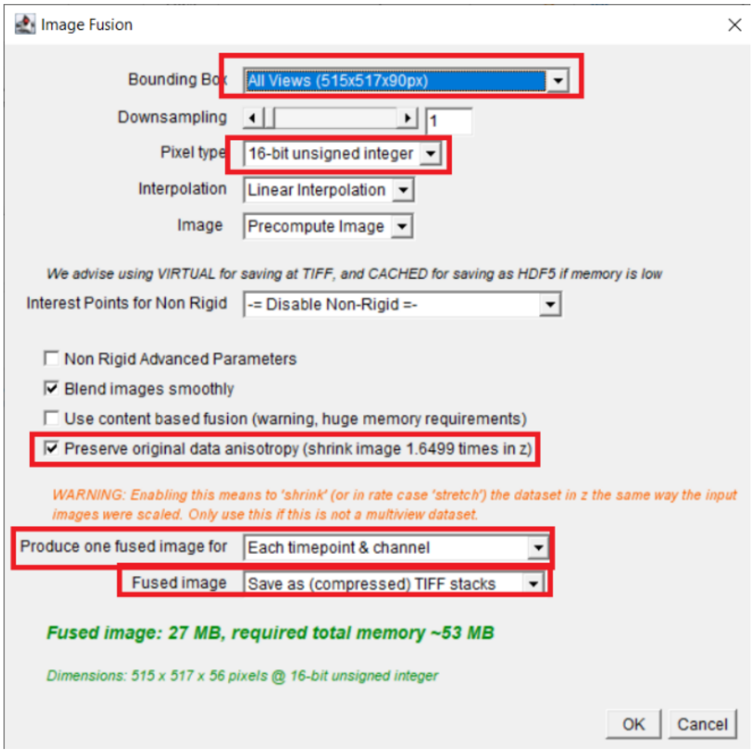

- Choose a separate folder for the registered stacks (do not use zip compression). The registered stacks will be saved to separate files for each channel and timepoint with a file name "fused_tp_XX_ch_XX.tif"

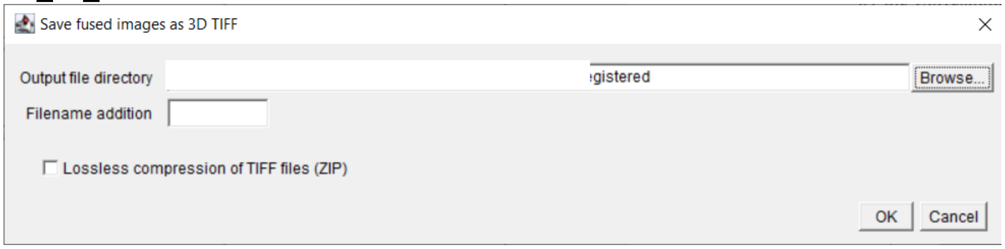

- The registration process is now completed, and every window associated with BigStitcher can now be closed.
- To assemble the registered stacks to the original 4D structure, drag and drop the "Assemble_4D_Stack" macro on the main FIJI window then press run. Select the folder where the registered stacks are stored, and press Run in the macro editor. This will assemble each image channel separately and the user will then be prompted to merge the channels.
- The denoised 4D stack is now registered and can be saved on the disk as a TIF file.

3.6 Registration of the Original Noisy 4D Stack using Pre-computed Transformations

- To validate the denoising performance it is necessary to compare the registered-denoised stack to a ground truth stack obtained by averaging the noisy data. Before doing that, however, it is necessary to register the noisy 4D stack using the same transformations that were previously computed. This procedure is also valid for registering stacks denoised with other algorithms.
- Open the noisy 4D stack in FIJI and use again the "Split_Timepoints" macro to save the single time points stack to a new folder.
- Open the folder where the BigStitcher dataset was saved (most likely the one containing the non-registered stacks), it should contain a subfolder named "interestpoints" and one or more "dataset.xml" files.

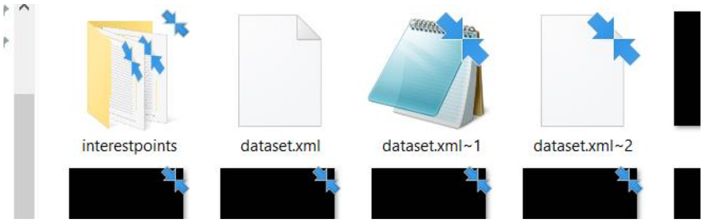

- Copy the interestpoints folder and the .xml files to the folder with the stacks to register.
- In FIJI open BigStitcher by clicking Plugins→BigStitcher→BigStitcher
- Browse to the location of the files to register and open the dataset.xml, the program should confirm the successful parsing of all the expected files.

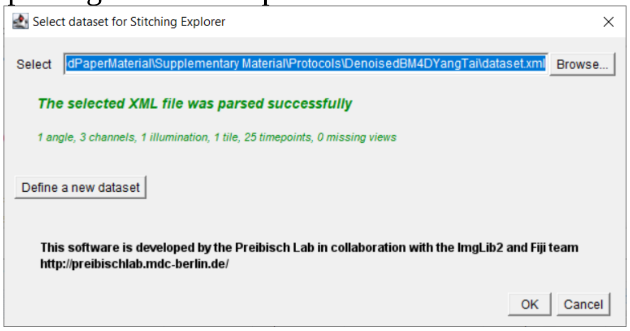

- The Multiview Explorer window will now appear. Follow the steps in section 3.5 to obtain a noisy-registered 4D stack.

4 DENOISING VALIDATION

- The plugin "SNR, PSNR, RMSE, MAE" at http://bigwww.epfl.ch/sage/soft/snr/ will be used to quantify the denoising performance against the ground truth stack. The plugin must be downloaded and installed in the plugins folder within the main FIJI folder. Restart the program after copying the file, the plugin will be available in Plugins→SNR.

- The quantitative metrics are measured on a slice-by-slice basis for each channel separately and are then averaged to get a mean PSNR for each channel.

4.1 Data trimming

- Registered data may have some “empty slices” and black borders, this occurs because the registration algorithm needs to shift each focal plane and stack to match the same structures over time (See figure below). This is a problem since the denoising performance metric (PSNR) will not provide reliable data if those areas are kept. All stacks should be trimmed to a common volume of interest containing only data.

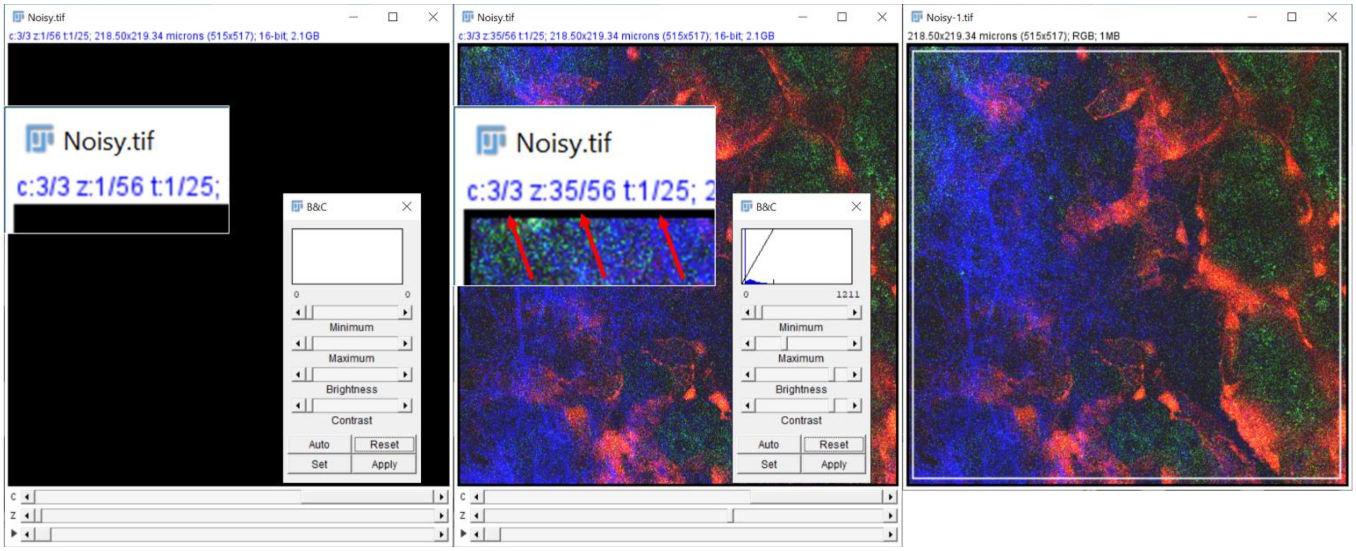

- Open the registered noisy 4D stack
- Browse the entire 4D stack in time and along the z planes, find the first and last slice that are present in all the time points (i.e. a common volume) and note them down.
- Draw a rectangular region of interest (ROI) and verify that the inner area does not contain any black border space, adjust the ROI as needed then press “t”. This will open the ROI Manager window and the ROI should appear in the list.

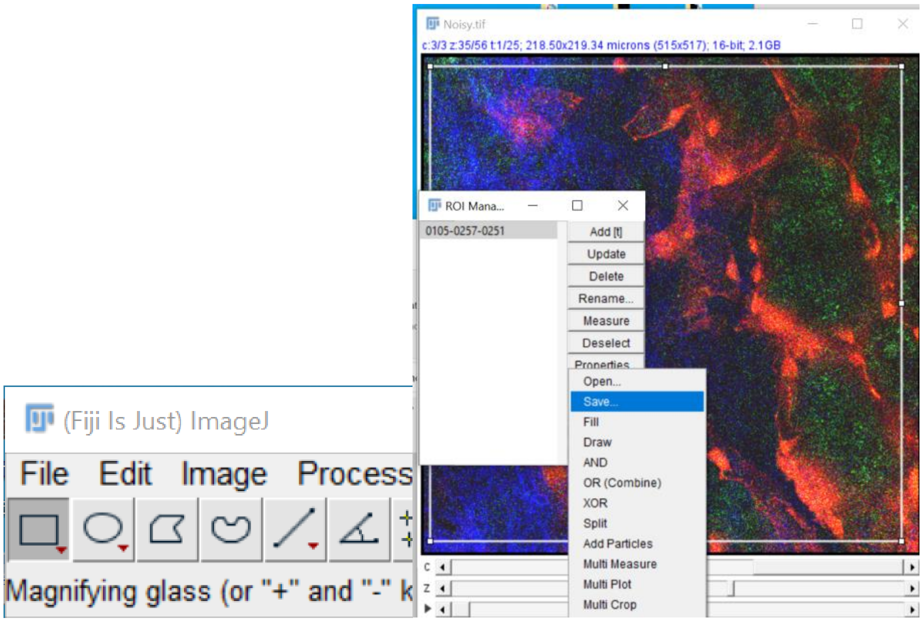

- In the ROI manager, select the ROI and then save it to a .roi file by clicking More→Save. Use a suggestive file name containing which z slices were previously noted to contain a common volume over time.
- Trim in 3D the 4D stack by selecting the ROI and then clicking on the 4D stack, then in FIJI use the command Image→Duplicate. Rename with an appropriate name (e.g. using a “noisy-trimmed”) and input the first and last slide to be included in the new trimmed stack then press OK.

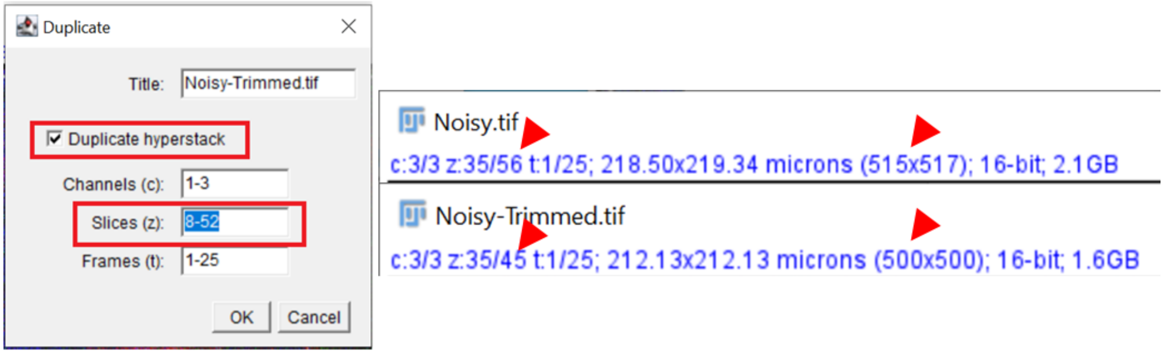

- The new trimmed 4D stack should now be slightly smaller (smaller number of z slices and pixels in xy). Save it to a new file in TIF format.
- All the stacks processed with different algorithms and registered in BigStitcher must be trimmed using the saved .roi file and by trimming on the same slices as it was just illustrated.

4.2 Ground Truth Generation through Averaging from Registered Noisy Data

- The ground truth is obtained by averaging the same slice over multiple noisy acquisitions to greatly reduce the effect of noise.
- Open the trimmed noisy 4D stack and drag on the main FIJI window the GroundTruthAverageNoisyTimePoints macro file then press “Run” in the “Macro editor” window.
- The new ground truth stack will now appear and show a clean 3D stack. This cannot be directly used for validation as the noisy and denoised stacks are 4D (i.e. they have more than

one timepoint). It is necessary to concatenate the ground truth stack to itself to create a 4D ground truth stack:

- Drag and drop on the main FIJI window the “GroundTruthConcatenate” and press “Run” in the “macro editor” window. The program will ask how many time points should be included in the 4D ground truth stack, in this case the validation data has 25.
- Save the 4D Ground truth stack as a tif file.

4.3 Quantitative Validation of the Denoising Performance Against a Ground Truth Stack

- Open in FIJI the 4D ground truth stack and the stack treated with algorithm to be validated.
- The ground truth and validation stacks must have the same dimensions (pixels and number of slices, time points).
- Split each stack into separate channels, select the stack and then Image→Colors→Split Channels
- Click on Plugins→SNR→SNR (Installation instructions are at the beginning of section 4 of the protocol). In the “Reference” field select a ground truth channel (e.g. Ch1) by clicking on “Change” and choosing the correct file. In the Test field choose the corresponding Noisy/Denoised channel then click on “Compute” button.
- The SNR window should now be populated by multiple values which can be copy/pasted in a spreadsheet for averaging or further data analysis.

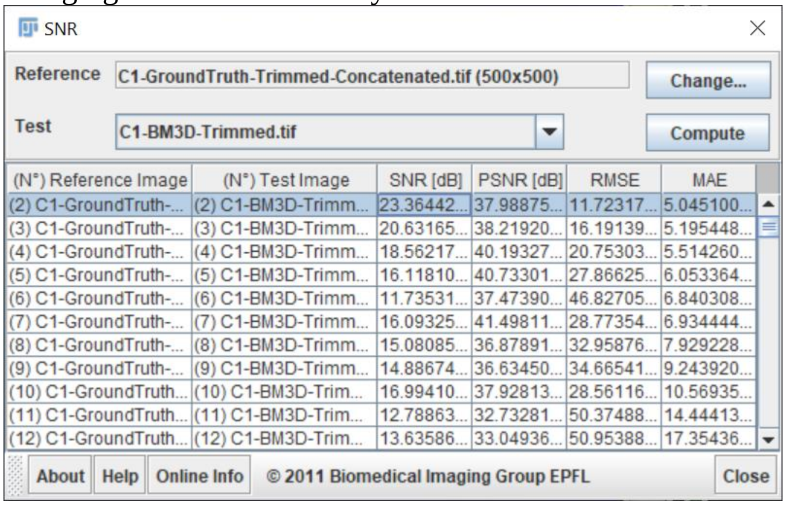

(N°) Reference Image	(N°) Test Image	SNR [dB]	PSNR [dB]	RMSE	MAE
(2) C1-GroundTruth-...	(2) C1-BM3D-Trimm...	23.36442...	37.98875...	11.72317...	5.045100...
(3) C1-GroundTruth-...	(3) C1-BM3D-Trimm...	20.63165...	38.21920...	16.19139...	5.195448...
(4) C1-GroundTruth-...	(4) C1-BM3D-Trimm...	18.56217...	40.19327...	20.75303...	5.514260...
(5) C1-GroundTruth-...	(5) C1-BM3D-Trimm...	16.11810...	40.73301...	27.86625...	6.053364...
(6) C1-GroundTruth-...	(6) C1-BM3D-Trimm...	11.73531...	37.47390...	46.82705...	6.840308...
(7) C1-GroundTruth-...	(7) C1-BM3D-Trimm...	16.09325...	41.49811...	28.77354...	6.934444...
(8) C1-GroundTruth-...	(8) C1-BM3D-Trimm...	15.08085...	36.87891...	32.95876...	7.929228...
(9) C1-GroundTruth-...	(9) C1-BM3D-Trimm...	14.88674...	36.63450...	34.66541...	9.243920...
(10) C1-GroundTruth...	(10) C1-BM3D-Trim...	16.99410...	37.92813...	28.56116...	10.56935...
(11) C1-GroundTruth...	(11) C1-BM3D-Trim...	12.78863...	32.73281...	50.37488...	14.44413...
(12) C1-GroundTruth...	(12) C1-BM3D-Trim...	13.63586...	33.04936...	50.95388...	17.35436...

- Close the SNR window and repeat the previous steps in the SNR plugin for each channel. The plugin window must be closed as changing the target images will not update the values.

5 REFERENCES

- **FIJI:** Schindelin, J., Arganda-Carreras, I., Frise, E., Kaynig, V., Longair, M., Pietzsch, T., … Cardona, A. (2012). Fiji: an open-source platform for biological-image analysis. Nature Methods, 9(7), 676–682. doi:10.1038/nmeth.2019
- **BigStitcher:** Hörl, D., Rojas Rusak, F., Preusser, F. et al. BigStitcher: reconstructing high-resolution image datasets of cleared and expanded samples. Nat Methods 16, 870–874 (2019). https://doi.org/10.1038/s41592-019-0501-0
