# Supplementary material for: Serial intravital 2-photon microscopy and analysis of the kidney using upright microscopes
Source: Front Physiol. 2023 Apr 24;14:1176409. doi: 10.3389/fphys.2023.1176409 (PMC10164931; doi:10.3389/fphys.2023.1176409)
Supplement: Supplementary file 3 [file DataSheet4.ZIP › IVM-Processing-Toolbox-Manual.pdf]

IVM Processing Toolbox Manual

1 General Instructions

- Follow the setup instructions in the setup guide.
- The provided scripts can be launched in FIJI by going to Plugins→IVM-Processing. The "Batch" scripts are intended for batch processing of entire datasets contained in a folder.

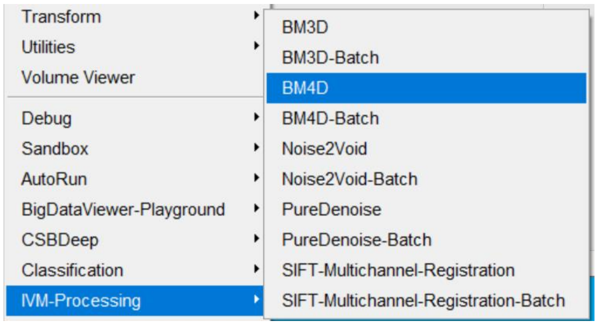

- Batch processing scripts for processing entire datasets in an input folder use the BioFormats reader and should be widely compatible with many microscopy formats, but slight code modifications could be necessary for specific use cases.
- The code has been tested on single images (t and z=1) or 3D stacks. 4D stacks may need to be split into separate time points (use the provided macro for the denoising validation protocol).
- The execution of the scripts can be interrupted by pressing the "ESC" key.

2 PureDenoise Image Denoising

2.1 PureDenoise – Single Images or Stacks

- Open the Image to denoise in FIJI and run Plugins→IVM-Processing→PureDenoise

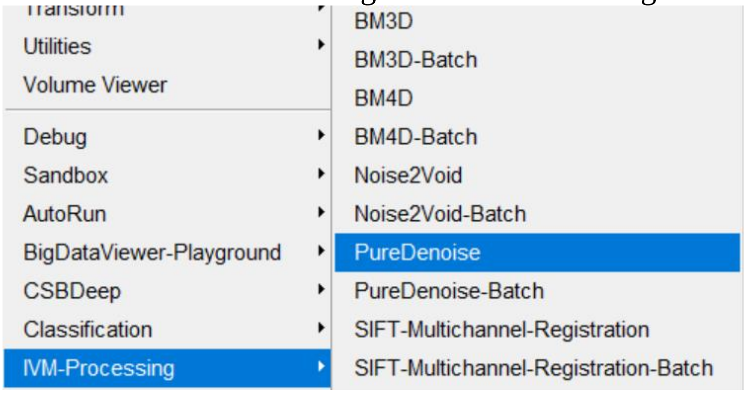

- The noisy image will disappear, and processing will begin. An ImageJ window will now appear overlaid to FIJI. This is an expected behavior.

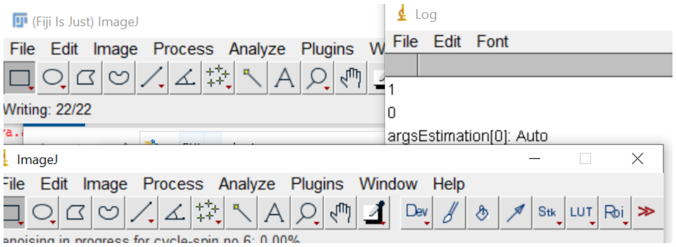

- Once denoised, the denoised image will appear next to the noisy image.

2.2 PureDenoise – Batch Denoising on Folders

- In FIJI got to Plugins→IVM-Processing→PureDenoise-Batch
- A dialog window will appear to ask for an input directory containing the noisy data, an output directory containing the denoised processed images, and the extension of the files to process (e.g. .czi, .lei, .oir, etc.). Press "OK" and wait, once the processing has been completed, a dialog window will be shown.

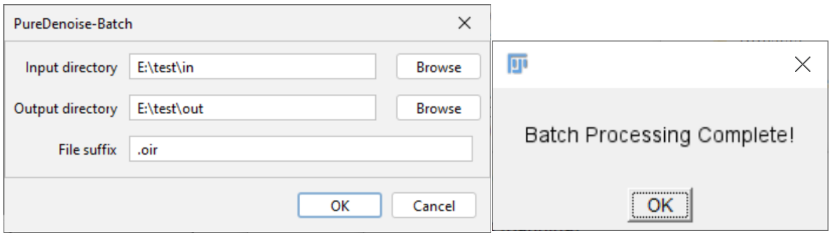

3 BMxD I

3.1 BMxD – Single Images or Stacks

- Open the Image to denoise in FIJI and run Plugins→IVM-Processing→BMxD (BM3D for images having z and t=1; BM4D for ZStacks or TSeries)
- The image will disappear, wait for the computation to occur (it may take several minutes for large stacks). Once denoised, the denoised image will appear next to the noisy image.

3.2 BMxD – Batch Denoising on Folders

- In FIJI go to Plugins→IVM-Processing→BMxD-Batch (BM3D for images having z and t=1; BM4D for ZStacks or TSeries).
- A dialog window will appear to ask for an input directory containing the noisy data, an output directory containing the denoised processed images, and the extension of the files to process (e.g. .czi, .lei, .oir, etc.). Press "OK" and wait, once the processing has been completed, a dialog window will be shown.

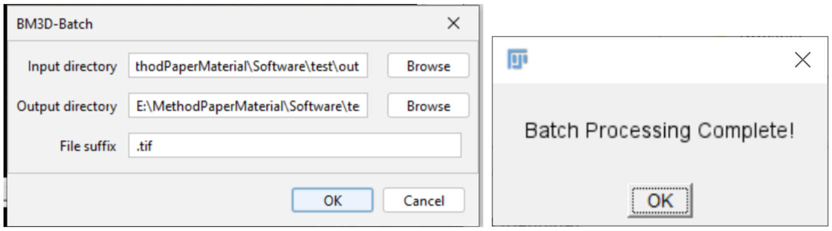

- BM4D may use a lot of RAM, if that becomes a problem, it may be useful to decrease the number of parallel workers in Matlab (please refer to the Matlab documentation).

- In case of datasets with mixed 3D data and 2D (z and t=1) data the two scripts for BM3D and BM4D must be executed in successive steps, as the BM3D script will ignore 3D data and vice versa for the BM4D one.

4 Noise2Void Image Denoising

4.1 Noise2Void – Training

- Noise2Void needs to be trained on noisy raw data. For intravital microscopy datasets with potentially thousands of single focal planes, we found that it is sufficient to select a representative subset of stacks for a total of 500-1000 slices.
- **The raw data needs to be acquired in a consistent fashion. E.g. it is not advisable to mix stacks having different detector gains!**
- Create a folder for each channel in the raw data (E.g. Ch1-Raw, Ch2-Raw, Ch3-Raw, …).
- Open each stack/image and split its channels Image→Split Channels and save each channel to its appropriate folder.
- In FIJI open Plugins→CSBDeep→N2V→Train on folder. Choose the folder containing the training data and the one containing the Validation data. If the same folders are used for training and validation, N2V will reserve a subset of image patches for validation. Check the "Use 3D model instead of 2D" to use a Noise2Void3D architecture which will have higher denoising performance but much higher computational and memory requirements.
- Training in the manuscript was done using two presets:
  - Noise2Void3D (epochs=300, steps=200, batch size=16, patch shape=48, neighborhood radius=5)
  - Noise2Void2D (epochs=300, steps=200, batch size=64, patch shape=64, neighborhood radius=5)

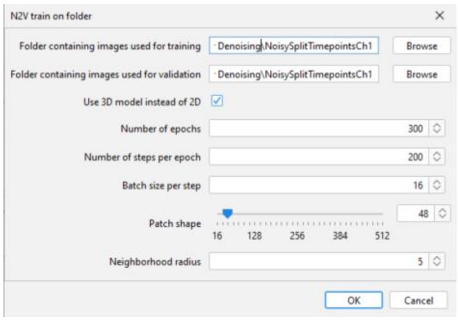

- Press "OK", the training will begin.

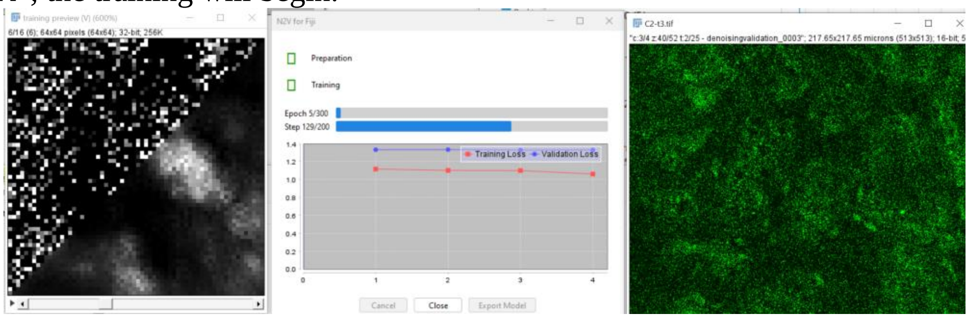

- Once the training is completed, two windows will appear asking whether to save the network with the lowest loss function score or the latest checkpoints. The Noise2Void documentation recommends saving the network at the latest iteration.

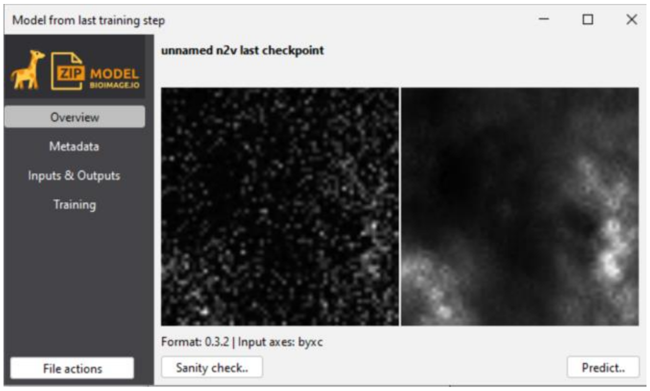

- Click on File actions and then on "Save to". Save the trained network in a useful location while maintaining the ".bioimage.io.zip" extension.

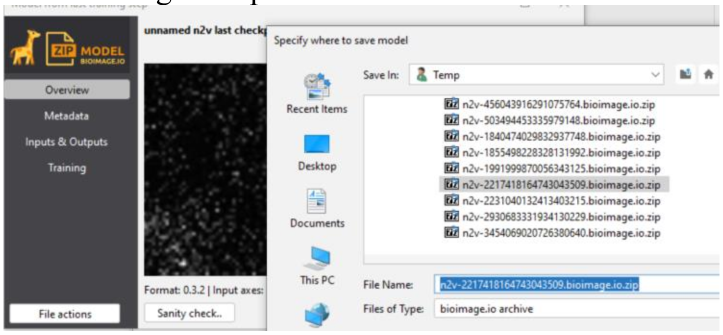

- The denoising script will use the trained network for each channel to denoise raw noisy data.
- Repeat the training process for all the channels.
- Noise2void can also be used on single stacks or images, use Plugins→CSBDeep→N2V→N2V-Train or Train and predict.

4.2 Noise2Void – Denoising Single Images or Stacks

- Open the Image to denoise in FIJI and run Plugins→IVM-Processing→Noise2Void. A dialog window will appear, set the path to the folder containing the trained networks, specify whether they have a 2D or 3D architecture and the number of channels of the image to process (up to 4). Then press "OK".

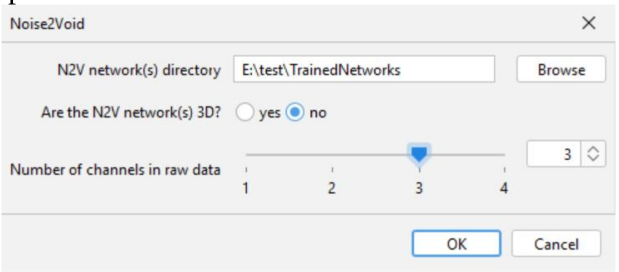

- In the next window specify which trained network to use for each channel then press "OK".

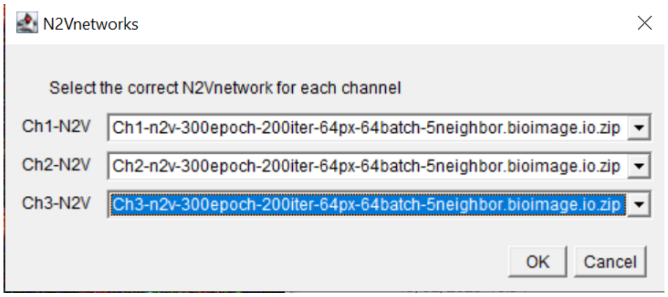

- The noisy image will disappear, and processing will begin. Once denoised, the denoised image will appear next to the noisy image.

4.3 Noise2Void – Batch Denoising on Folders

- In FIJI go to Plugins→IVM-Processing→ Noise2Void-Batch.
- A dialog window will appear to ask for an input directory containing the noisy data, an output directory containing the denoised processed images, a directory containing the trained networks, the extension of the files to process (e.g. .czi, .lei, .oir, etc.), the architecture of the trained neural networks (2D or 3D) and the number of channels contained in the data (up to 4). Specify all the parameters and press “OK”. After all the data has been processed, a dialog window will appear to provide confirmation.

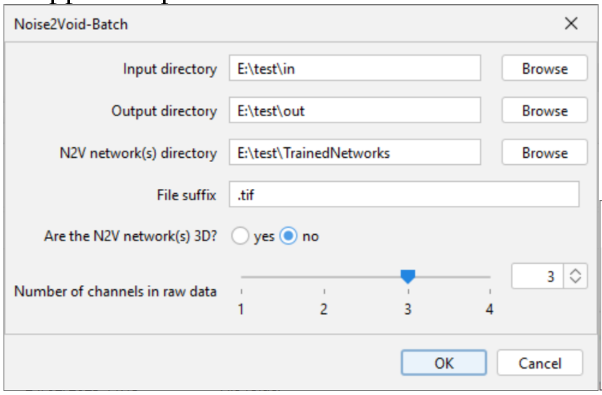

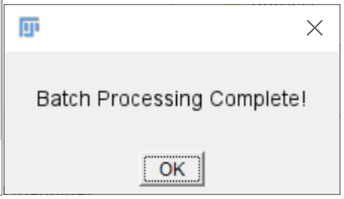

5 SIFT Image Registration to Compensate for Sample Drift

5.1 SIFT – Registration of Single Stacks

- Open the Image to register in FIJI and run Plugins→IVM-Processing→SIFT-Multichannel-Registration
- A dialog window will ask for the parameters used by the SIFT algorithm. The default values have been tested on the two included test stacks and provide good starting values. It might be necessary to adjust them. See below for more in-depth documentation. Answer “yes” if the stack to register is mostly empty in the first slices. Press "OK” once all the SIFT parameters have been set up.

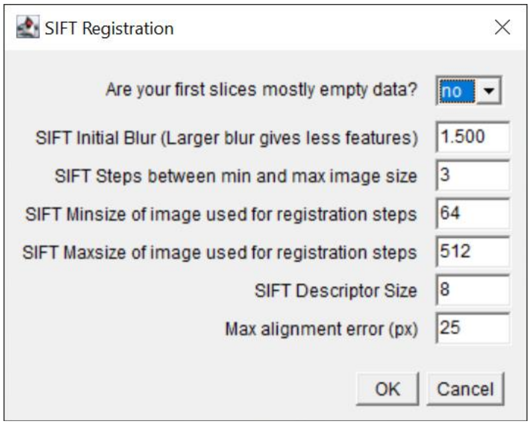

- The image will disappear, wait for the computation to occur. Once registered, the aligned image will appear next to the raw image.

5.2 SIFT – Batch Registration on Folders

- In FIJI go to Plugins→IVM-Processing→ SIFT-Multichannel-Registration-Batch
- A dialog window will appear to ask for an input directory containing the data affected by sample drift, an output directory containing the registered data, and the extension of the files to process (e.g. .czi, .lei, .oir, etc.). A second dialog where the SIFT parameters can be adjusted will then appear (see previous section), press "OK" and wait, once the processing has been completed, a dialog window will be shown.

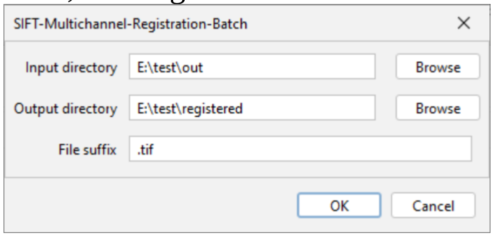

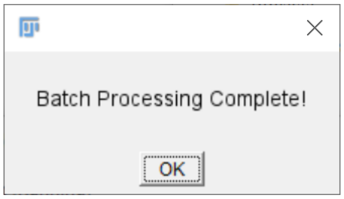

5.3 SIFT – Tuning of Registration Parameters

- The default SIFT parameters have been tested on the two provided sample stacks, but the registration performance may prove unsatisfactory when used on different data. In this case a fine tuning of the SIFT parameters may be necessary.
- On typical 512x512 px data, these settings should result in 50-100+ common image features between neighboring frames. This should provide good registration performance without excessive computational load.

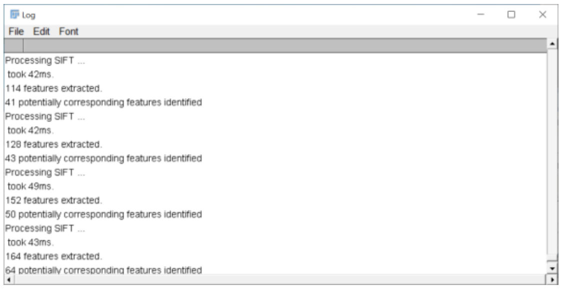

- See documentation at https://imagej.net/plugins/feature-extraction for the Scale Invariant Interest Point Detector documents each setting.
- In case of registration failure, it may be beneficial to use a lower initial blur value, higher minimal image size, more SIFT steps or a combination of all the above. There is no benefit in using a maximum size that is larger than the image size (i.e. >512 for a 512x512px stack)

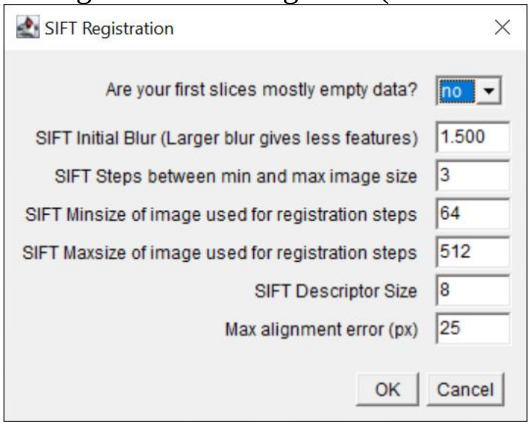

6 REFERENCES

6.1 FIJI & ImageJ

- **ImageJ:** Schneider, C. A., Rasband, W. S., & Eliceiri, K. W. (2012). NIH Image to ImageJ: 25 years of image analysis. Nature Methods, 9(7), 671–675. doi:10.1038/nmeth.2089
- **FIJI:** Schindelin, J., Arganda-Carreras, I., Frise, E., Kaynig, V., Longair, M., Pietzsch, T., … Cardona, A. (2012). Fiji: an open-source platform for biological-image analysis. Nature Methods, 9(7), 676–682. doi:10.1038/nmeth.2019

6.2 PureDenoise

- F. Luisier, C. Vonesch, T. Blu, M. Unser, "Fast Interscale Wavelet Denoising of Poisson-corrupted Images", Signal Processing, vol. 90, no. 2, pp. 415-427, February 2010.
- F. Luisier, "The SURE-LET Approach to Image Denoising", Swiss Federal Institute of Technology Lausanne, EPFL Thesis no. 4566 (2010), 232 p., January 8, 2010.
- F. Luisier, C. Vonesch, T. Blu, M. Unser, "Fast Haar-Wavelet Denoising of Multidimensional Fluorescence Microscopy Data", Proceedings of the Sixth IEEE International Symposium on Biomedical Imaging: From Nano to Macro (ISBI'09, Boston MA, USA, June 28-July 1, 2009, pp. 310-313.

6.3 BMxD

- **BM3D:** K. Dabov, A. Foi, V. Katkovnik and K. Egiazarian, "Image Denoising by Sparse 3-D Transform-Domain Collaborative Filtering," in *IEEE Transactions on Image Processing*, vol. 16, no. 8, pp. 2080-2095, Aug. 2007, doi: 10.1109/TIP.2007.901238.
- **BM4D:** M. Maggioni, V. Katkovnik, K. Egiazarian, A. Foi, "A Nonlocal Transform-Domain Filter for Volumetric Data Denoising and Reconstruction", IEEE Trans. Image Process., vol. 22, no. 1,pp. 119-133, Jan. 2013. doi:10.1109/TIP.2012.2210725
- **Noise Estimation:** An implementation of noise estimation according to S.-M. Yang and S.-C. Tai:"Fast and reliable image-noise estimation using a hybrid approach" Journal of Electronic

Imaging 19(3), pp. 033007-1--15, 2010. Implentation released by Chris Schwemmer, Universität Erlangen-Nürnberg https://www5.cs.fau.de/en/our-team/schwemmer-chris/software/index.html

- **Generalized Anscombe Transformation:** Generalized Anscombe VST (J.L. Starck, F. Murtagh, and A. Bijaoui, Image Processing and Data Analysis, Cambridge University Press, Cambridge, 1998)
- **GAT inversion:**
    - M. Makitalo and A. Foi, "Optimal Inversion of the Generalized Anscombe Transformation for Poisson-Gaussian Noise," in IEEE Transactions on Image Processing, vol. 22, no. 1, pp. 91-103, Jan. 2013, doi: 10.1109/TIP.2012.2202675.
    - M. Makitalo and A. Foi, "A Closed-Form Approximation of the Exact Unbiased Inverse of the Anscombe Variance-Stabilizing Transformation," in IEEE Transactions on Image Processing, vol. 20, no. 9, pp. 2697-2698, Sept. 2011, doi: 10.1109/TIP.2011.2121085.

6.4 Noise2Void

- A. Krull, T. -O. Buchholz and F. Jug, "Noise2Void - Learning Denoising From Single Noisy Images," 2019 IEEE/CVF Conference on Computer Vision and Pattern Recognition (CVPR), Long Beach, CA, USA, 2019, pp. 2124-2132, doi: 10.1109/CVPR.2019.00223.
- Weigert, M., Schmidt, U., Boothe, T. *et al.* Content-aware image restoration: pushing the limits of fluorescence microscopy. *Nat Methods* **15**, 1090–1097 (2018). https://doi.org/10.1038/s41592-018-0216-7
