# Supplementary material for: Serial intravital 2-photon microscopy and analysis of the kidney using upright microscopes
Source: Front Physiol. 2023 Apr 24;14:1176409. doi: 10.3389/fphys.2023.1176409 (PMC10164931; doi:10.3389/fphys.2023.1176409)
Supplement: Supplementary file 3 [file DataSheet4.ZIP › IVM-Processing-Toolbox-Setup.pdf]

Supplementary Material

IVM Processing Toolbox Setup

1 FIJI Installation and Setup

- Download FIJI at https://fiji.sc/ and unzip the FFIJI.app folder on the Desktop.
- Open the FIJI folder

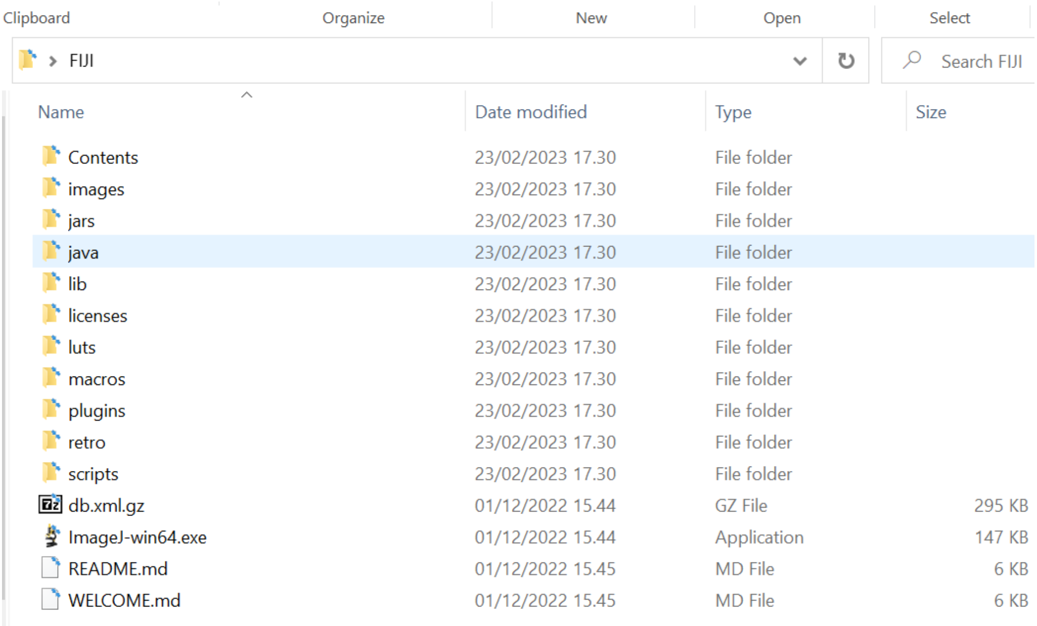

- Copy the provided "IVMdenoising" folder to the FIJI folder

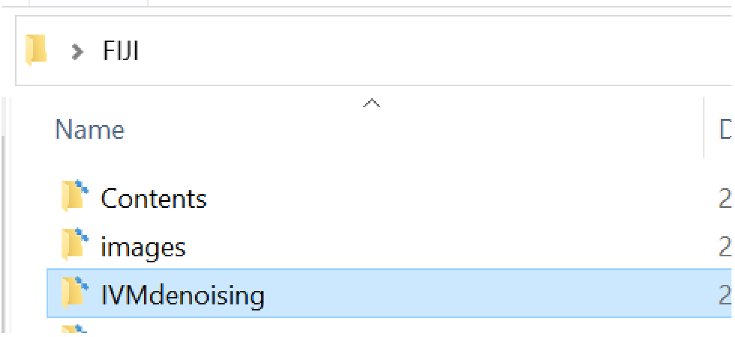

- Copy the provided "IVM-Processing_ folder" in the FIJI/plugins subfolder.

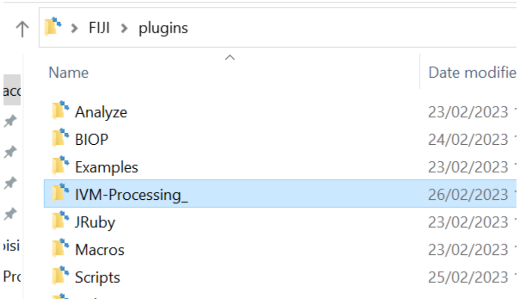

- Download ImageJ at https://imagej.nih.gov/ij/download.html and open the zip file.

- Copy the ImageJ folder within the archive inside FIJI/IVMdenoise subfolder.

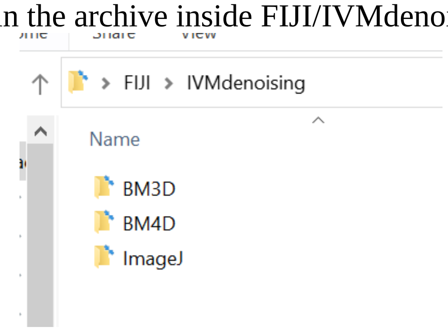

1.1 Updating FIJI and installing the necessary update sites.

- Start FIJI and in the top menu go to Help→Update, wait for the updater to find new software versions and the ImageJ Updater window should appear. Cick on Manage Update Sites.

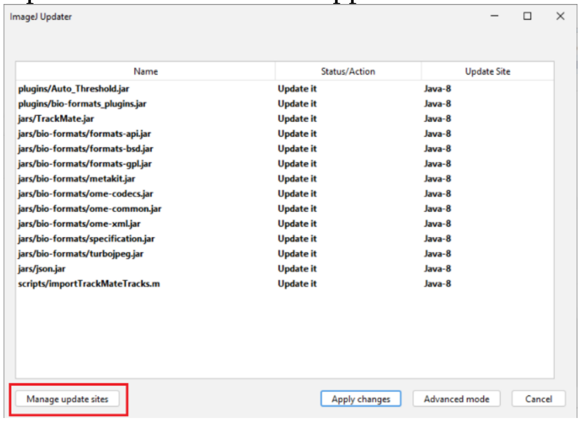

- Select the following update sites: "CSB-Deep, Tensorflow, PTBIOP" then click on "Close" and then on "Apply Changes" in the Updater window.

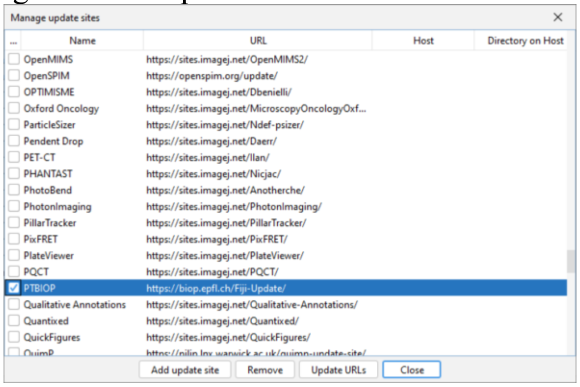

- Wait for the installation to complete, close FIJI and restart the program. You may need to repeat the procedure 2-3 times to have a completely up to date FIJI installation.

2 PureDenoise Setup

- Section 1 provides the necessary steps to follow before installing PureDenoise.
- Copy the "PureDenoiseIJ.jm" macro from the provided archive to FIJI/IVMdenoising/ImageJ/Plugins/

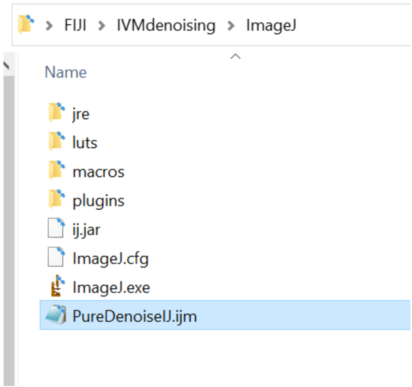

- Download PureDenoise at http://bigwww.epfl.ch/algorithms/denoise/ . Download both the distribution for end-users and the one for macro or development.

Download and install

Distribution for end-users	Distribution for macro or development
Download the ImageJ plugin **PureDenoise_.jar**.	Download **PureDenoise.zip**, the ImageJ's plugin.
Put the file PureDenoise_.jar in the *plugins* folder of ImageJ without unzip it.	Unzip the file PureDenoise.zip and put it into the *plugins* folder of ImageJ.<br>**Macro:** The syntax of the macro is described in this following example **Macro-Example-PureDenoise.txt**.
The JAR file includes the Java classes, sources, and documentation.	The ZIP file includes the Java classes, sources, and documentation.

- Copy the PureDenoise_.jar file in the FIJI/IVMdenoising/ImageJ/Plugins/ subfolder..
- Open the downloaded PureDenoise.zip and copy the PureDenoise_ folder in the FIJI/IVMdenoising/ImageJ/Plugins/ subfolder.

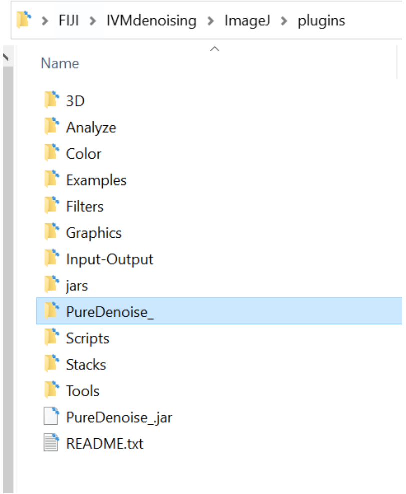

- Optional: also copy the PureDenoise_.jar file in the FIJI/Plugins folder. This will be useful to test different frames and cycle settings on single isolated image channels. PureDenoise cannot be scripted from within FIJI due to the removal of the javac (Java compiler from the main FIJI distribution). Javac is still bundled to ImageJ, as a workaround to allow the use of PureDenoise scripts from within FIJI, the code will create temporary files on disk to denoise data in ImageJ and then read back the cleaned image in FIJI for visualization.

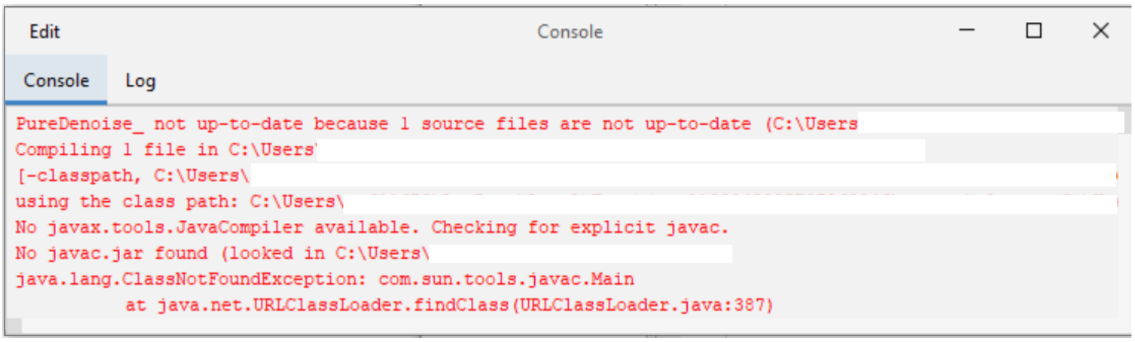

3 BMxD Setup

- BMxD algorithms are executed within the Matlab environment, it is assumed that the user has a working Matlab installation with the "Image Processing Toolbox" and the "Parallel Computing Toolbox" installed. For information on the setup please refer to https://www.mathworks.com/help/matlab/matlab_env/get-add-ons.html .
- Section 1 provides the necessary steps to follow before installing BMxD filters.
- Download BM3D and BM4D from https://webpages.tuni.fi/foi/GCF-BM3D/

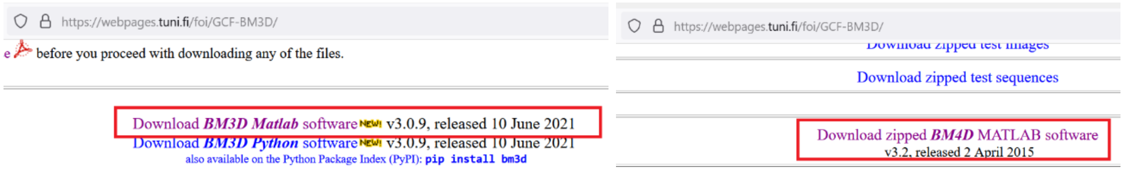

- Open the BM3D zip archive and copy the contents of the bm3d subfolder in the zip archive to the FIJI/IVMdenoising/BM3D/BM3D subfolder.

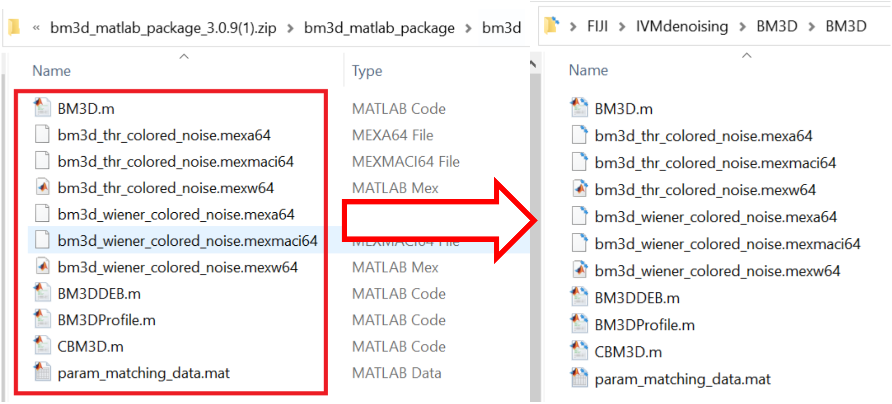

- Open the BM4D zip archive and copy the contents to the FIJI/IVMdenoising/BM4D/BM4D subfolder.

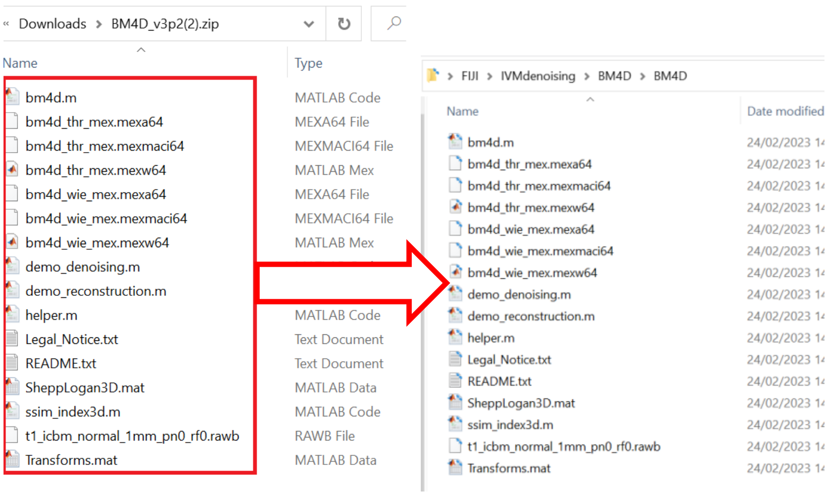

4 Noise2Void Setup

- Section 1 provides the preliminary steps to follow to install CSB-Deep and Tensorflow in FIJI.
- It is assumed that the user has an Nvidia GPU and is using Windows. Linux distributions and MacOS were not tested.
- Install The Nvidia libraries "**CUDA 10**" and the compatible "**cudnn**" (7.6.5.32). They are hosted in the archive section of the Nvidia website and it is necessary to create an account to download cudnn. At the time of writing, the official Imagej-Tensorflow wiki at https://imagej.net/develop/tensorflow recommends installing CUDA 10.0 and then adding multiple parameters to the system's path, but our system worked without them. The latest CUDA version (12.0 at this time) was tested and found to be incompatible with the FIJI plugins for N2V.

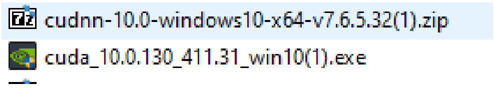

- Start FIJI and go to Edit→Options→Tensorflow. The default version should be TF 1.12.0 CPU, select 1.15.0 GPU. Other GPU-accelerated versions may also work. The software will download the necessary components and ask to restart FIJI. Close the program.

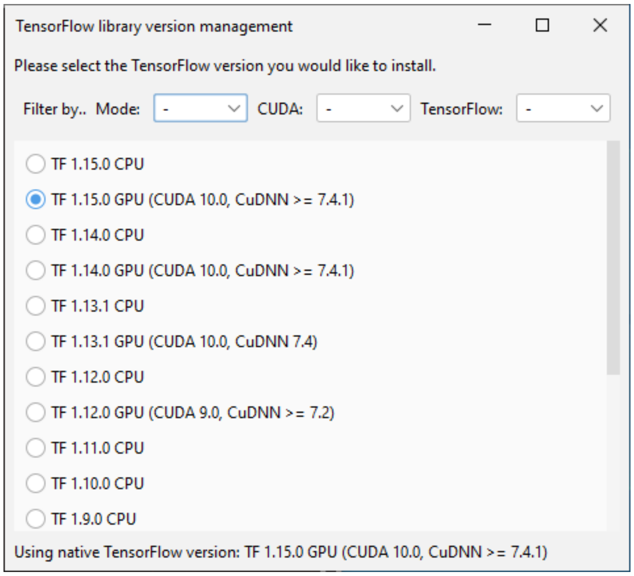

- **Do not restart FIJI right away.** Open the FIJI folder and the FIJI/update/lib/win64 then copy the tensorflow_jni.dll to the main FIJI folder. This operation is currently needed due to a bug in FIJI (https://forum.image.sc/t/imagej-tensorflow-now-with-command-to-change-tensorflow-library-version-switch-to-gpu/31744/6). If FIJI was restarted by accident the TF version will be back to 1.12.0 CPU, switching again to TF 1.15.0GPU will download again the tensorflow.jni.dll file.

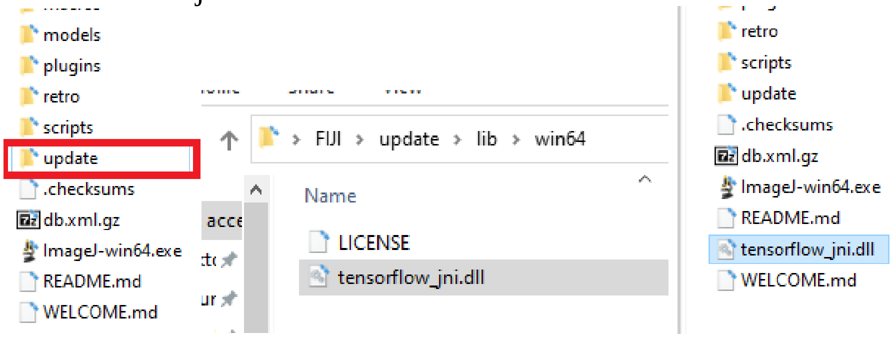

- Start FIJI and open a noisy image, then go to Edit→Options→Tensorflow, the program should display that the previously selected “GPU” version is selected.
- To verify that everything is correctly working open a sample image, split its channels if needed and then go to Plugins→CSBDeep→N2V→N2V Train

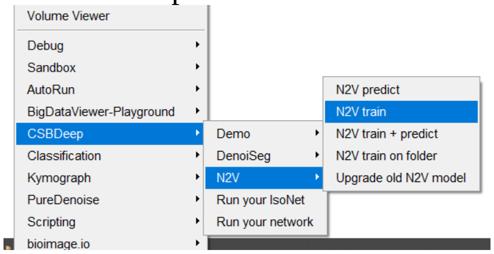

- In the training window leave everything to default and press OK. The training of the neural network should now begin. Open the Windows Task Manager, if CPU utilization is extremely high while the training steps are increasing slowly (4-5s per step) this means that the setup was not successful.

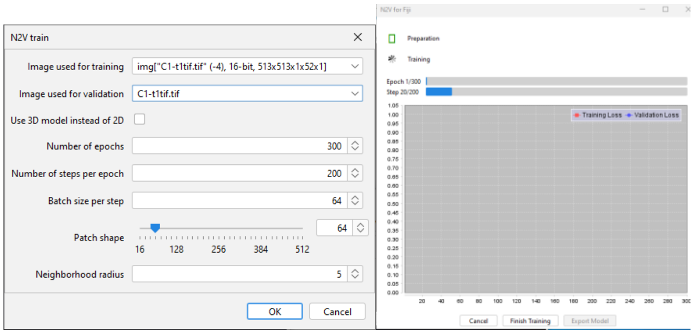
N2V train
Image used for training
img["C1-t1tif.tif" (-4), 16-bit, 513x513x1x52x1]
Image used for validation
C1-t1tif.tif
Use 3D model instead of 2D
Number of epochs
300
Number of steps per epoch
200
Batch size per step
64
Patch shape
64
16
128
256
384
512
Neighborhood radius
5
OK
Cancel
N2V for Fiji
Preparation
Training
Epoch 1/300
Step 20/200
Training Loss
Validation Loss
Cancel
Finish Training
Export Model
