# Supplementary material for: Serial intravital 2-photon microscopy and analysis of the kidney using upright microscopes
Source: Front Physiol. 2023 Apr 24;14:1176409. doi: 10.3389/fphys.2023.1176409 (PMC10164931; doi:10.3389/fphys.2023.1176409)

3D-Printed Mouse Holder Assembly Instructions

1 Materials

- 3D-printed holder components (2 x Vertical pillars, 2 x Horizontal Supports, 2 x Lateral Clamps, 1 x Top Plate, 1 x Central plate, 2 x Locking Clips
- 10 x M6 1.5cm bolts
- 6 x M6 nuts
- 2 x M6 8cm bolts
- 2 x M6 joining nuts or thumb nuts.

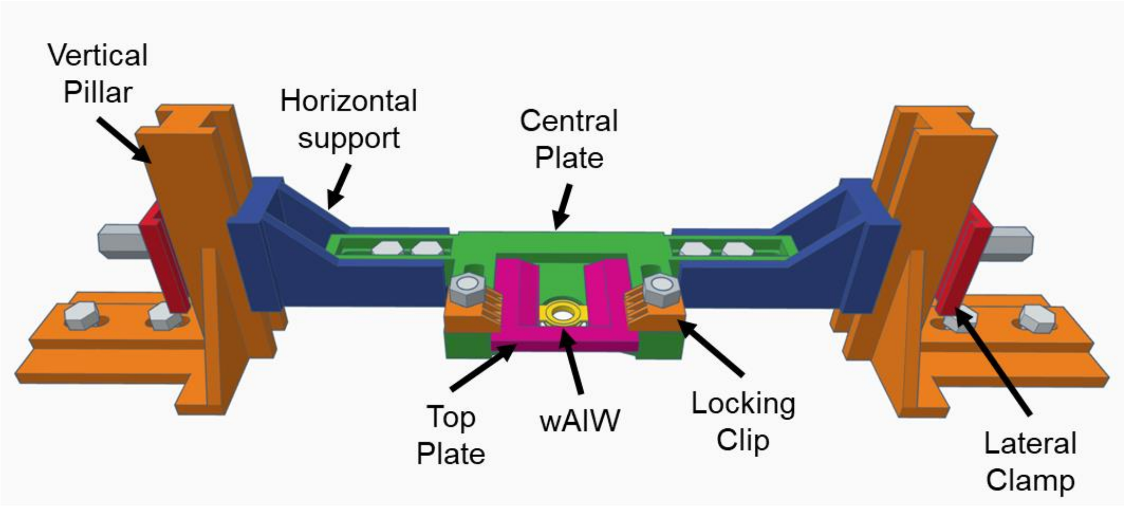

2 Assembly Instructions

- 3D print the plastic components. In the manuscript we used a Creality Ender 3 Pro mounting a 0.4mm nozzle and PLA filament. ABS should probably work. Set the printer slicer to 0.2mm layer height and high-density infill (60% in our case) to produce strong parts with limited flexing.
- Begin by assembling the horizontal holders to central plate by inserting 4 x M6 1.5cm bolts with the threaded side facing down then tighten an M6 nut to each bolt. The central plate has a housing which allows the bolts to slide freely and adjust the width of the holder. This allows the user to make the necessary adjustments to fit the width of their own setup (e.g. heating pads).

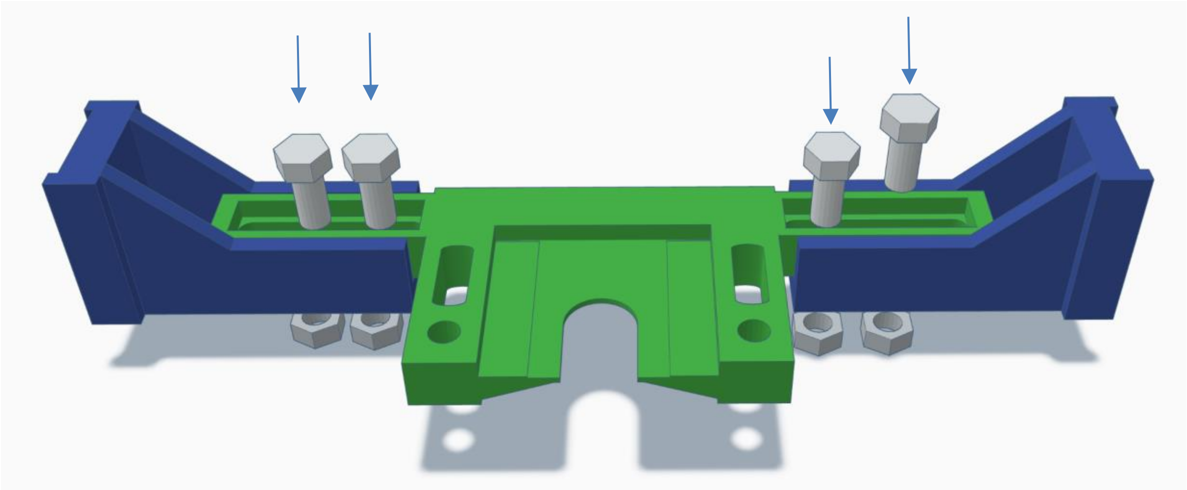

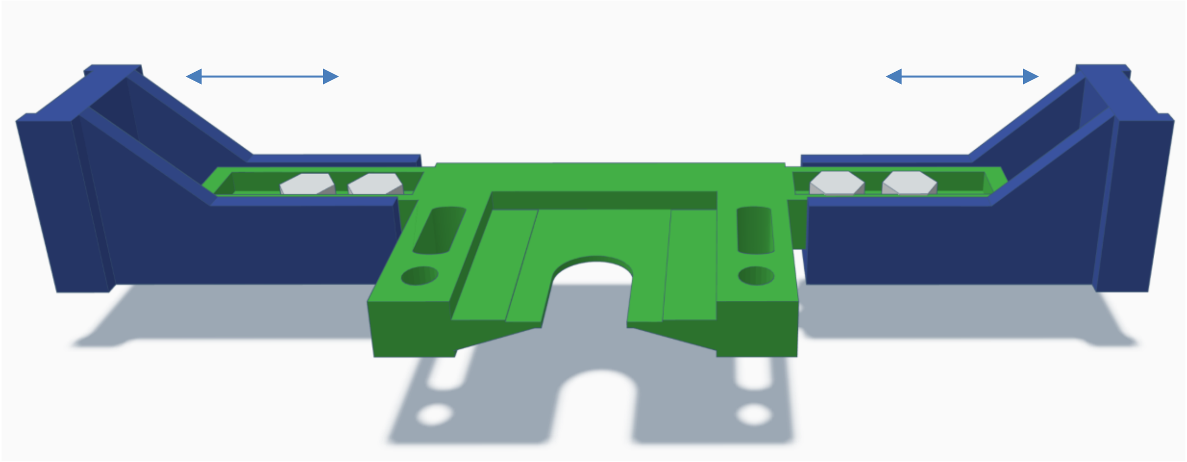

- Thread a M6 8cm through the side housing in the lateral holder.

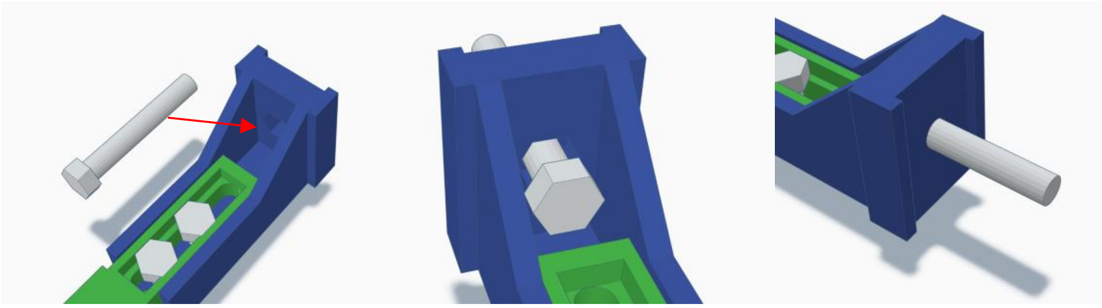

- Insert the protruding nut in then empty vertical rail within a pillar.

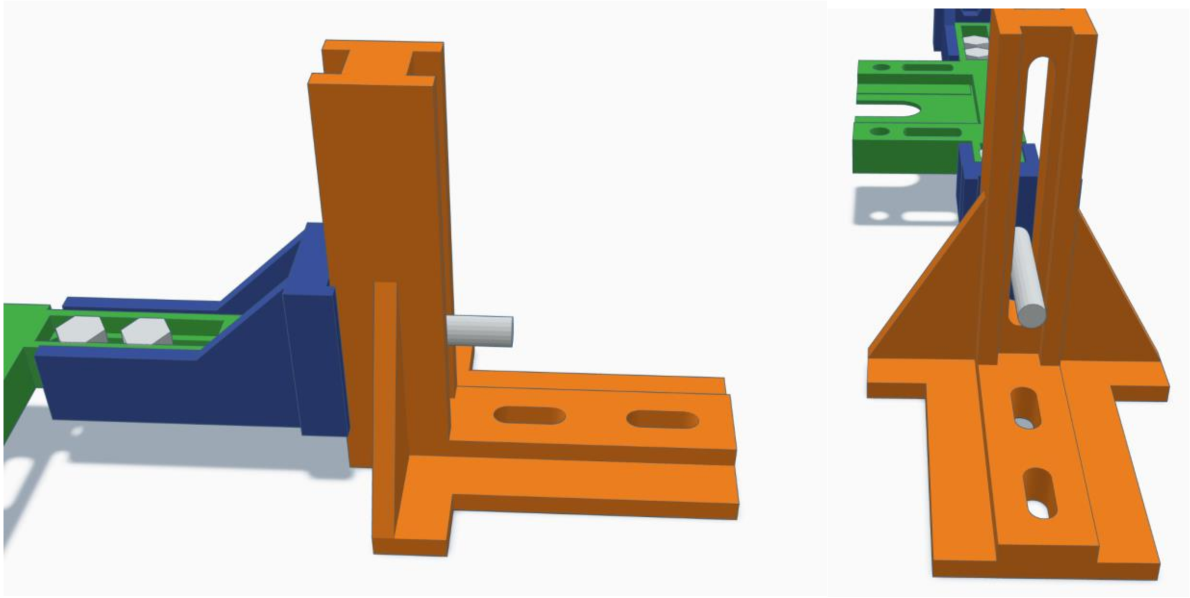

- Mount a lateral plate through its hole and tighten it to the pillar with an M6 joining nut.

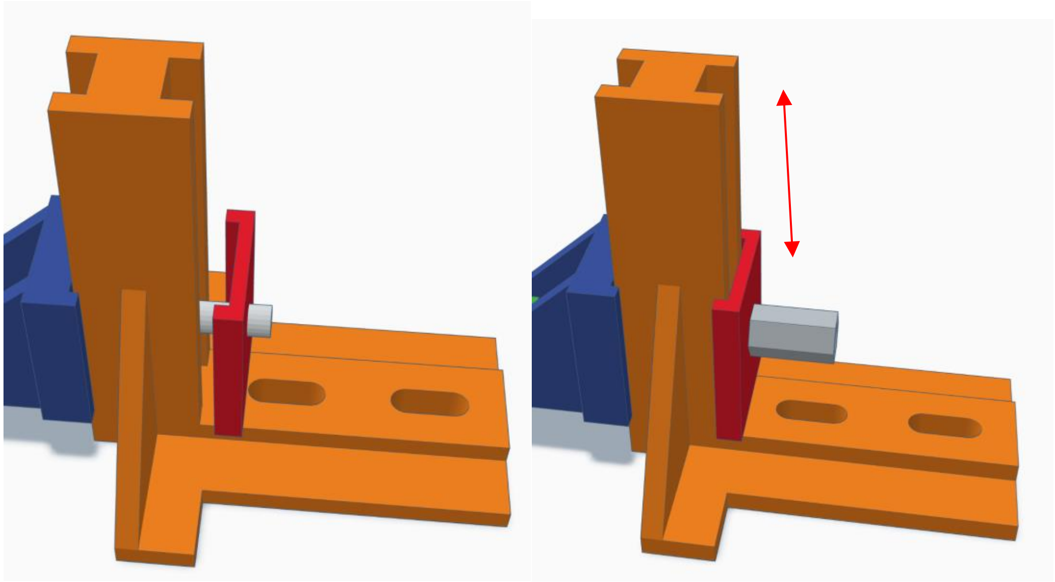

- Repeat the same last steps to assemble the second Vertical Pillar. The pillars provide a way to adjust the height of horizontal plate to fit animals of different sizes or to adapt the holder to different setups. The joining nuts provide a better grip to tighten the clamp, but a normal M6 nut may work too. A small water scale placed on the horizontal plate can assist with maintaining a constant horizontal orientation of the base plate after changing the height at each side.
- To mount the lateral clips that will press down the top plate to the base plate, begin by inserting an M6 1.5cm bolt from below in one of the two hexagonal housings on the lower side of the base plate. Press the bolt up with a finger and insert a clip, then tighten an M6 bolt. Then repeat the operation on the other side.

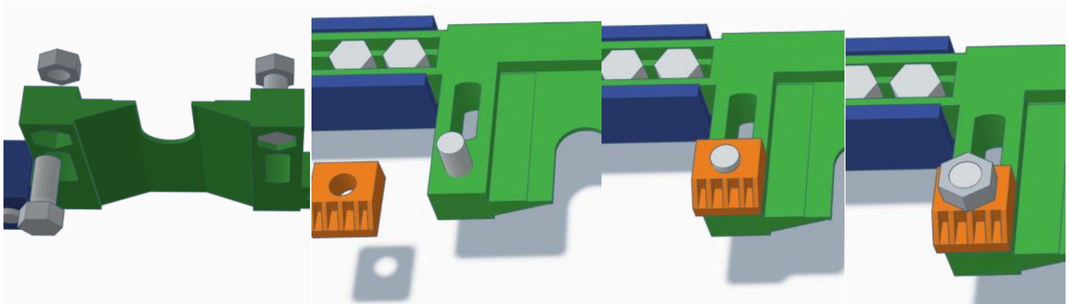

- The remaining 4 M6 screws should be used to lock the holder to the microscope stage. In the absence of threaded holes in the stage, adhesive tape may be used to stabilize the setup while imaging.

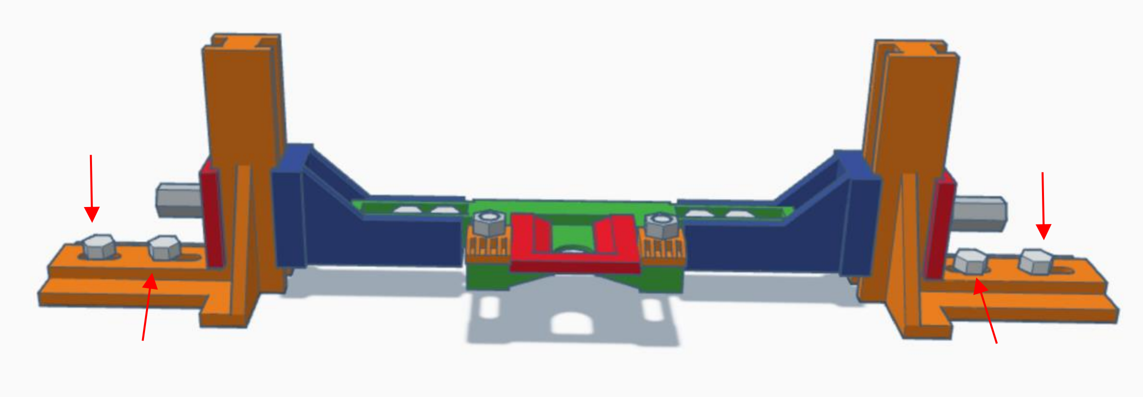

Supplement: Supplementary file 4 [file DataSheet1.ZIP › 3D printed holder-Assembly Instructions.pdf]
