# Supplementary figures and images for: Serial intravital 2-photon microscopy and analysis of the kidney using upright microscopes
Source: Front Physiol. 2023 Apr 24;14:1176409. doi: 10.3389/fphys.2023.1176409 (PMC10164931; doi:10.3389/fphys.2023.1176409)

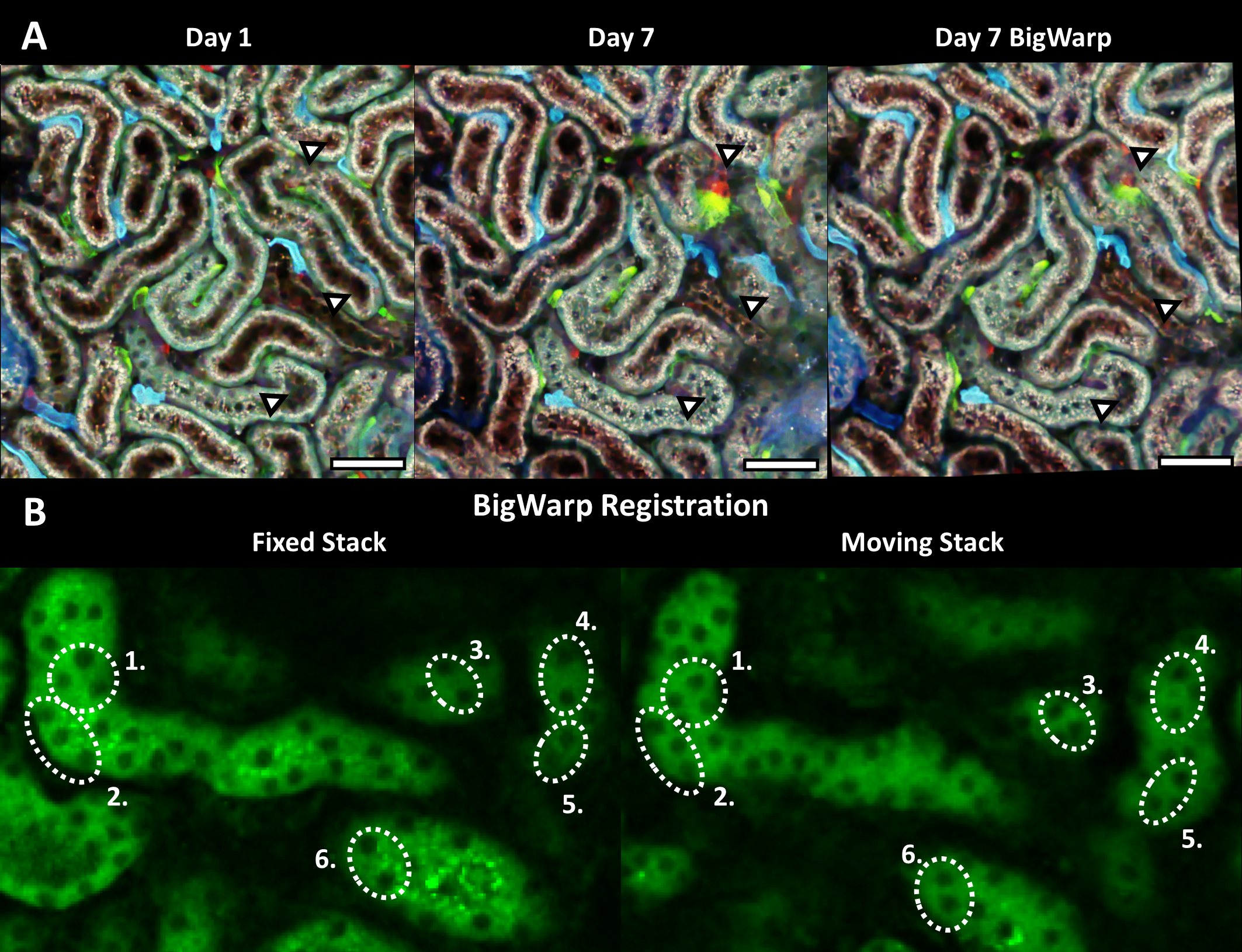

Supplement: Supplementary file 5 [file Image2.tif]

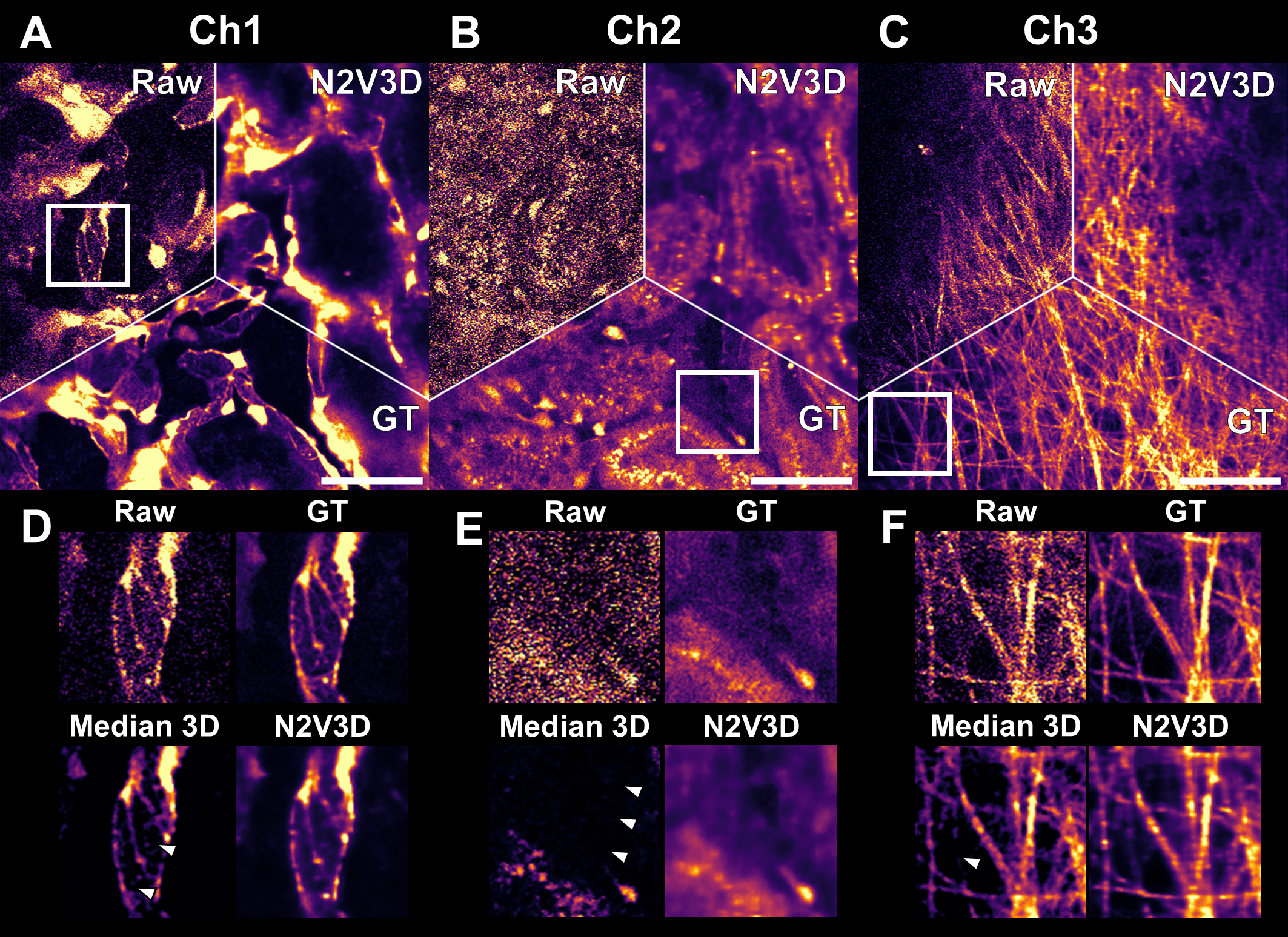

Supplement: Supplementary file 6 [file Image1.tif]
